# DualPG‐DTA: A Large Language Model‐Powered Graph Neural Network Framework for Enhanced Drug‐Target Affinity Prediction and Discovery of Novel CDK9 Inhibitors Exhibiting In Vivo Anti‐Leukemia Activity

**DOI:** 10.1002/advs.202513099

**Published:** 2026-01-27

**Authors:** Yihao Chen, Jindi Huang, Cong Liu, Shipeng Zhang, Xinze Li, Zhang Zhang, Tie‐Gen Chen, Ling Wang

**Affiliations:** ^1^ Joint International Research Laboratory of Synthetic Biology and Medicine Ministry of Education Guangdong Provincial Key Laboratory of Fermentation and Enzyme Engineering Guangdong Provincial Engineering and Technology Research Center of Biopharmaceuticals School of Biology and Biological Engineering South China University of Technology Guangzhou 510006 China; ^2^ State Key Laboratory of Bioactive Molecules and Druggability Assessment School of Pharmacy Jinan University Guangzhou 510632 China; ^3^ International Cooperative Laboratory of Traditional Chinese Medicine Modernization and Innovative Drug Discovery of Chinese Ministry of Education Guangzhou City Key Laboratory of Precision Chemical Drug Development School of Pharmacy Jinan University Guangzhou 510632 China; ^4^ Zhongshan Institute for Drug Discovery Shanghai Institute of Materia Medica Chinese Academy of Sciences Zhongshan 528400 China; ^5^ Shanghai Institute of Materia Medica Chinese Academy of Sciences Shanghai 201203 China

**Keywords:** CDK9, inhibitor, Drug‐target binding affinity prediction, graph neural networks, pre‐training, virtual screening

## Abstract

Accurate prediction of drug‐target interactions constitutes a crucial foundation for drug discovery. DualPG‐DTA is presented, a general framework for binding affinity prediction that integrates two pre‐trained language models to generate atomic‐level molecular representations and residue‐level protein embeddings. The architecture constructs dual molecular‐protein graphs processed through dedicated graph neural networks equipped with dynamic attention mechanisms to extract context‐aware sequence‐level features, which are fused via a multimodal module for affinity predictions. Benchmark results show that DualPG‐DTA consistently outperforms existing models across all metrics. Applied to CDK9 inhibitor discovery, the framework is used to develop robust regression/classification models and identified compound **C1** as a novel CDK9 inhibitor with an IC_50_ of 1.2 nM. **C1** demonstrates exceptional CDK family selectivity alongside optimal pharmacokinetic properties, including prolonged half‐life, adequate clearance, robust plasma exposure, and oral bioavailability. Notably, oral **C1** demonstrated potent antitumor efficacy in a Venetoclax‐resistant MV4‐11 acute myeloid leukemia (AML) xenograft model, with concurrent demonstration of favorable tolerability and safety profiles. Collectively, the study not only establishes a unified framework for precise binding affinity prediction but also identifies **C1** as a highly promising therapeutic lead targeting CDK9 to conquer Venetoclax resistance in AML.

## Graphical abstract

This study introduces DualPG‐DTA, a framework integrating two pre‐trained models to generate molecular and protein representations. It constructs dual graphs processed by specialized neural networks with dynamic attention for feature fusion, achieving superior benchmark performance. Applied to CDK9 inhibitor discovery, it identifies compound **C1**—a potent, selective inhibitor with excellent efficacy and safety in resistant AML models.

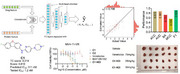

## 1 Introduction

The use of traditional biochemical and molecular biology methods to evaluate drug‐target interaction strength requires expensive reagents, specialized equipment, and resource‐intensive experimental preparation coupled with complex data analysis. In contrast, deep learning (DL) approaches can automatically extract discriminative features from the structural or sequential information of drug molecules and target proteins to predict their binding affinities. These methodologies demonstrate particular efficacy in processing large‐scale biomedical data, offering transformative tools and innovative paradigms for drug discovery.^[^ ^1^ ^]^ In 2018, DeepDTA emerged as the first DL‐based method for predicting continuous drug‐target binding affinities.^[^ ^2^ ^]^ Unlike conventional computational approaches that perform binary classification of drug activity, DeepDTA employed novel convolutional neural networks (CNNs) to regress quantitative binding values. While utilizing simplified 1D representations of drugs and proteins as input, this architecture achieved superior performance compared to conventional machine learning (CML)‐based algorithms on large benchmark datasets. In 2019, an enhanced DeepDTA variant termed WideDTA^[^ ^3^ ^]^ was proposed. This framework employed a vocabulary‐based (vs. character‐based) strategy to integrate the sequence information of drug molecules and target proteins, processing textual information such as protein domains, functional motifs, and maximal common substructures of compounds through CNNs. WideDTA demonstrated superior performance over DeepDTA on two commonly used benchmark datasets (Davis and KIBA). Zhao et al. proposed AttentionDTA,^[^ ^4^ ^]^ implementing attention mechanisms to dynamically weight critical subsequences in both protein targets and ligand molecules, significantly improving prediction accuracy. Nguyen et al. advanced the field with GraphDTA,^[^ ^5^ ^]^ a new graph neural network (GNN)‐based architecture representing drug molecules as graphs for binding affinity prediction. Multimodal DL models can process and integrate multiple types of data (e.g., text, images, and sound). They combine information from different modalities to provide a more comprehensive understanding and analysis of the data, thereby improving prediction accuracy. Zhong et al. proposed MMDTA,^[^ ^6^ ^]^ a multimodal framework employing CNN‐GNN hybrid architectures to extract multidimensional heterogeneous features from drug‐target sequences and 3D structures. The model integrates these features via a mixed fusion strategy to generate drug‐target binding affinity predictions. Wang et al.^[^ ^7^ ^]^ introduced AffinityVAE, a multi‐objective DL framework that leverages a variational autoencoder combined with protein–ligand interaction feature mapping. This approach enables simultaneous prediction of binding affinity and generation of novel drug‐like molecules, while also offering interpretable visualizations of binding sites.

Pretrained models represent a paradigm in DL that expedites task‐specific learning and enhances predictive performance by capturing generalized feature representations from large datasets, thereby serving as a starting point for subsequent tasks. The knowledge acquired from pretraining on vast amounts of data can be transferred to new related tasks. For example, in natural language processing (NLP), models like BERT^[^ ^8^ ^]^ and GPT^[^ ^9^ ^]^ are pretrained on massive text corpora to capture deep semantic and structural features of natural language. In computer vision (CV), foundational models such as ResNet^[^ ^10^ ^]^ and VGGNet^[^ ^11^ ^]^ leverage large‐scale image datasets to learn common features and effectively recognize and classify objects in images. Within structural biology, AlphaFold^[^ ^12^ ^]^ exemplifies this paradigm through pretraining on extensive protein sequence data, 3D structural data, and evolutionary information, enabling accurate prediction of the 3D structures of novel proteins, which holds significant practical implications and application value in drug discovery and related fields.

In this study, we propose DualPG‐DTA, an innovative DL framework that synergistically integrates dual pretrained large language models with GNNs equipped with multi‐head attention mechanisms for drug‐target binding affinity prediction. We then conducted systematic benchmarking against state‐of‐the‐art CML and DL approaches spanning the past decade using two established benchmark datasets. The results demonstrated that DualPG‐DTA achieves optimal performance across all evaluation metrics. Furthermore, we extended DualPG‐DTA to the task of cyclin‐dependent kinase 9 (CDK9) inhibitor discovery and successfully identified compound **C1** as a novel CDK9 inhibitor. **C1** not only demonstrated potent inhibitory activity against CDK9 but also displayed favorable pharmacokinetic and safety properties in vivo. Additionally, it exhibited notable anti‐leukemia efficacy in a xenograft mouse model of MV4‐11‐VR that was resistant to the Bcl‐2 inhibitor Venetoclax.

## 2 Results and Discussion

### 2.1 Architecture and Performance of the DualPG‐DTA Model

**Figure 1:**
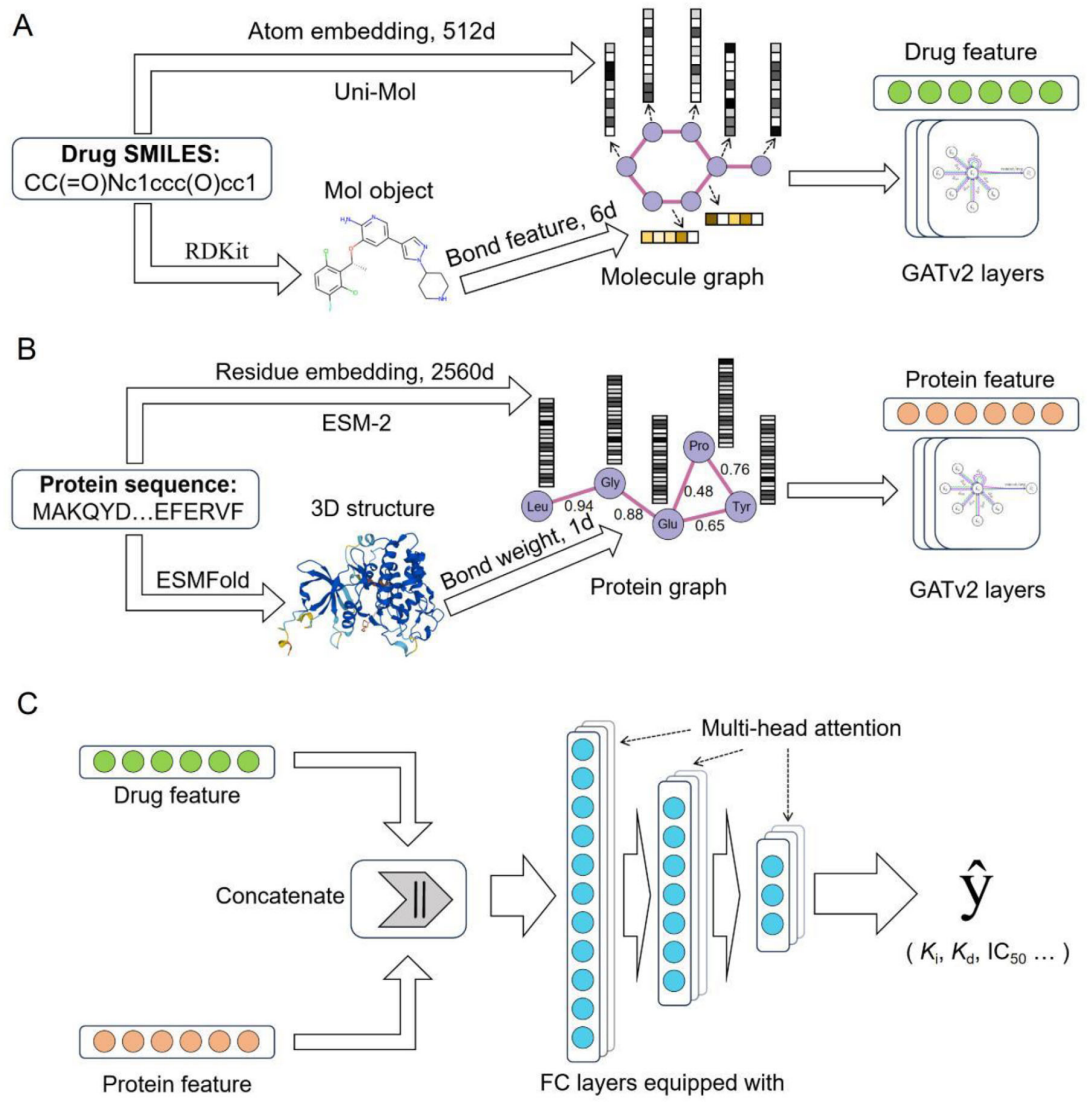
Architecture diagram of DualPG‐DTA. A) Drug feature extraction module. B) Protein feature extraction module. C) Fusion prediction module.

Accurate prediction of drug‐target interactions is fundamental for drug discovery. We introduce DualPG‐DTA, a deep learning framework designed to predict binding affinity by integrating two pre‐trained large language models. As illustrated in **Figure** **1**, its architecture comprises three core components: a drug feature extraction module, a protein feature extraction module, and a multimodal fusion prediction module. The core innovation lies in leveraging these pre‐trained models to capture atomic‐level 3D structural information of small molecules and residue‐level embeddings of proteins. This information is processed through GATv2 networks with dynamic attention mechanisms, which extract context‐aware sequence‐level features from dual molecular‐protein graphs. For drug graphs, GATv2 dynamically assigns higher attention weights to key pharmacophoric features such as aromatic rings and hydrogen bond donors by leveraging learned edge features (Figure 1A). For protein graphs, residues located within 8Å of the binding pocket—for example, the hinge region in CDK9 (Figure 3F)—are assigned prioritized attention through distance‐based edge weights. This dual‐graph attention mechanism enables the model to focus on structurally meaningful interactions. A multimodal fusion module then integrates these features to generate precise predictions of drug‐target binding affinity, enabling systematic exploration of complex interactions in drug discovery.

To comprehensively evaluate the performance of the DualPG‐DTA model, we trained the model and tested all evaluation metrics on two benchmark datasets (Davis and KIBA). The reported results are averaged from five independent experimental indicators of fivefold cross‐validation. As shown in **Table** **1**, our model achieved the best performance, with the highest Concordance Index (CI) and $r_{m}^{2}$ values of 0.908 and 0.788 for Davis and 0.894 and 0.791 for KIBA, and the lowest Mean Squared Error (MSE) values of 0.169 and 0.138 for Davis and KIBA, respectively. Specifically, compared with the previous best model (BiComp‐DTA), the MSE values of the DualPG‐DTA on the two datasets were reduced by 21.8% and 8.0%; the CI indexes of the DualPG‐DTA increased by 0.4% and 0.3% for Davis and KIBA, while the $r_{m}^{2}$ values of the DualPG‐DTA improved by 11.8% and 4.5% for Davis and KIBA, respectively. In addition, our model has achieved a significant advantage on the Davis dataset with less data, which is the advantage brought by pretrained models introduced upstream of the DualPG‐DTA. Because the pretrained models have learned a lot of general knowledge, when the amount of data for downstream tasks is limited, the pretrained models can adapt to new tasks faster than ordinary models, helping the model to grasp more general features, so that the model can learn more useful information with less data.

**Table 1:** Performance results of DualPG‐DTA model on Davis and KIBA datasets.

Methods	Davis			KIBA		
	MSE	C	$r_{m}^{2}$	MSE	CI	$r_{m}^{2}$
KronRLS^[^ ^13^ ^]^	0.379	0.871 (0.001)	0.407 (0.005)	0.411	0.782 (0.001)	0.342 (0.001)
SimBoost^[^ ^14^ ^]^	0.282	0.872 (0.002)	0.644 (0.006)	0.222	0.836 (0.001)	0.629 (0.007)
DeepDTA^[^ ^2^ ^]^	0.261	0.878 (0.004)	0.630 (0.017)	0.194	0.863 (0.002)	0.673 (0.009)
AttentionDTA^[^ ^4^ ^]^	0.216	0.893 (0.005)	0.677 (0.024)	0.155	0.882 (0.004)	0.755 (0.017)
MATT‐DTI^[^ ^15^ ^]^	0.227	0.891 (0.003)	0.683 (0.009)	0.150	0.889 (0.001)	0.756 (0.011)
GraphDTA^[^ ^5^ ^]^	0.258	0.884 (0.002)	0.656 (0.014)	0.162	0.879 (0.004)	0.736 (0.028)
FusionDTA^[^ ^16^ ^]^	0.220	0.903 (0.002)	0.666 (0.008)	0.167	0.890 (0.001)	0.699 (0.010)
BiComp‐DTA^[^ ^17^ ^]^	0.237	0.904 (0.001)	0.696 (0.012)	0.167	0.891 (0.001)	0.757 (0.012)
G‐K BertDTA^[^ ^36^ ^]^	0.267	0.879 (0.003)	0.660 (0.003)	0.201	0.847 (0.003)	0.675 (0.01)
TEFDTA^[^ ^19^ ^]^	0.264	0.878 (0.002)	0.635 (0.021)	0.199	0.857 (0.002)	0.703 (0.005)
AttentionMGT‐DTA^[^ ^20^ ^]^	0.193	0.891 (0.005)	0.699 (0.027)	0.140	0.893 (0.001)	0.786 (0.018)
InceptionDTA(CharVec)^[^ ^18^ ^]^	0.196	0.891 (0.003)	0.625 (0.020)	0.163	0.887 (0.001)	0.716 (0.018)
InceptionDTA(Seq)^[^ ^18^ ^]^	0.242	0.897 (0.002)	0.624 (0.028)	0.166	0.890 (0.001)	0.707 (0.007)
DualPG‐DTA	**0.169**	**0.908 (0.003)**	**0.778 (0.009)**	**0.138**	**0.894 (0.001)**	**0.791 (0.009)**

Note: Bolded values represent the optimal results for the metric. The values in parentheses represent the standard deviation of the cross‐validation process. The performance evaluation values for the baseline models (KronRLS, SimBoost, DeepDTA, AttentionDTA, MATT‐DTI, GraphDTA, FusionDTA, and BiComp‐DTA) were collected from the experimental data of Kalemati et al.^[^ ^17^ ^]^. The performance results for the remaining baseline models were sourced from Wu et al.^[^ ^20^ ^]^ and Kalemati et al.^[^ ^18^ ^]^.

Table 1 also shows that the two CML‐based models, KronRLS and SimBoost, generally perform worse than various DL models. The possible reason is that DL models can better adapt to the deep and complex feature representations in drug and protein data, and effectively handle the nonlinear relationship between drug and protein interactions. On the other hand, when both the drug and protein bilateral architectures are CNNs, DL models with attention mechanisms such as AttentionDTA and MATT‐DTI have significant performance improvements compared to the DeepDTA method without attention mechanisms. This is because the attention mechanisms can help the model adapt to different types of biological data in a more flexible way and focus on their key parts. It is worth noting that in terms of drug representation, GraphDTA, which uses GNNs for 2D molecular graph feature extraction, has a certain performance improvement over the DeepDTA model that only uses CNNs for 1D SMILES feature extraction. Collectively, our DualPG‐DTA model uses two pretrained large models and two sets of three‐layer GATv2 network modules as feature extractors for drugs and proteins, effectively integrating the advantages of pre‐training strategies, GAT, and attention mechanisms, ultimately leading to state‐of‐the‐art performance in drug‐target binding affinity prediction tasks.

Furthermore, to validate DualPG‐DTA, we benchmarked it against several recent methods—StructureNet,^[^ ^21^ ^]^ PLAIG,^[^ ^22^ ^]^ and GNNSeq^[^ ^23^ ^]^—using the PDBBind v2020 refined set, the same dataset adopted in these studies. As shown in Table S1 (Supporting Information), our model achieves the best mean squared error (MSE = 1.384) and mean absolute error (MAE = 0.951), along with a near‐optimal Pearson correlation coefficient (PCC = 0.776), underscoring its strong predictive accuracy.

### 2.2 Multi‐Level Model Validations

#### 2.2.1 Visualizinsg the Relationship between Predicted and Experimental Values

**Figure 2:**
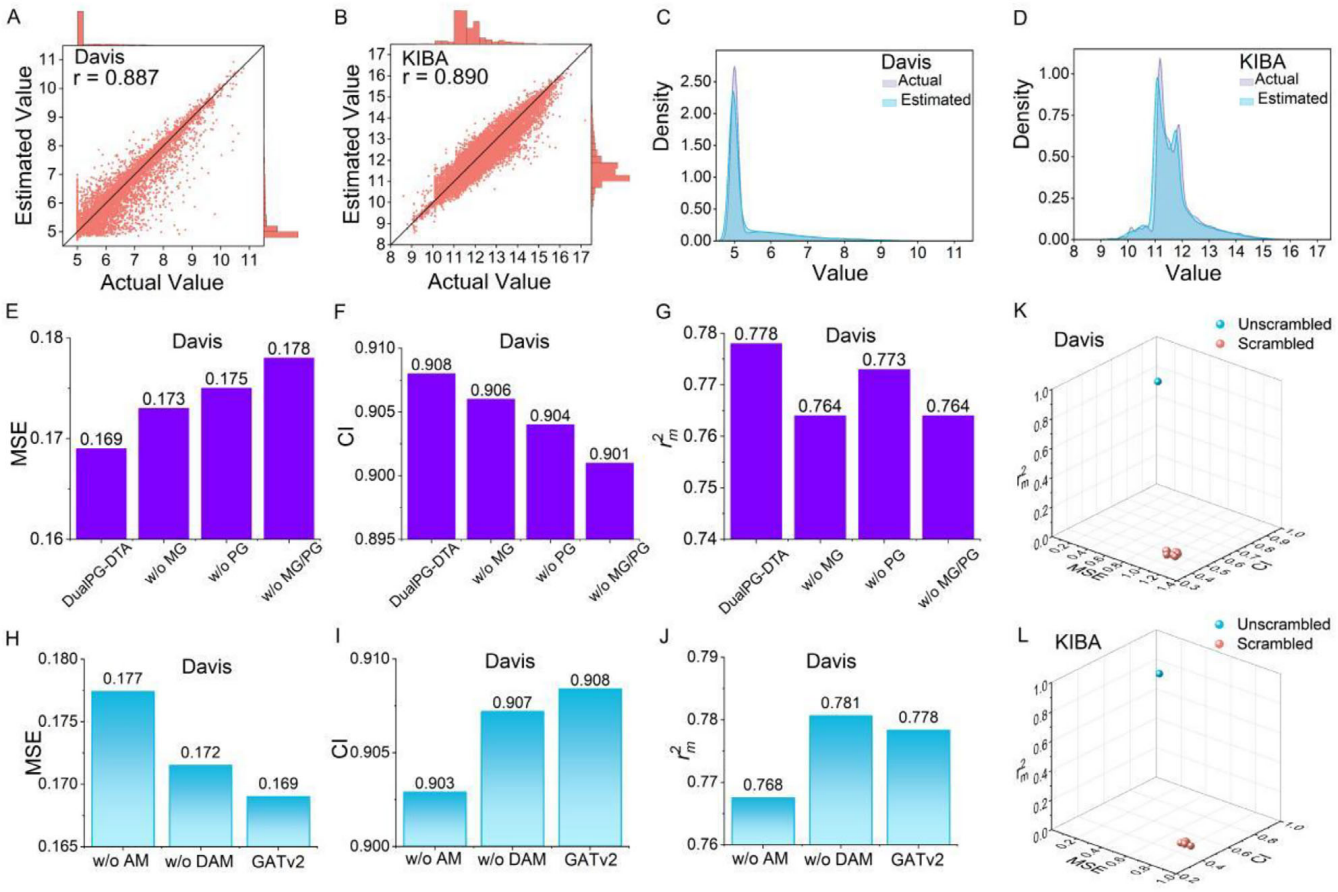
Multi‐level validation of the DualPG‐DTA predictive performance. Scatter density plots with marginal histograms for comparing experimental values vs estimated binding affinities of DualPG‐DTA on Davis (A) and KIBA (B) datasets. Kernel density estimation plot for actual value vs estimated value of DualPG‐DTA on Davis (C) and KIBA (D) datasets. Different input features ablation results of the models on the MSE (E), CI (F), and $r_{m}^{2}$ (G) metrics of Davis dataset. Different GNN variants ablation results of the models on MSE (H), CI (I), and $r_{m}^{2}$ (J) metrics of Davis dataset. Y‐Scrambling validation results of DualPG‐DTA on Davis (K) and KIBA (L) datasets.

Visualizing the relationship between predicted values and experimental (Actual) values is one of the important steps in evaluating model performance, because it can help us intuitively understand the prediction accuracy and deviation of the model. As shown in **Figure** **2**A,B, the distribution of the predicted values is closely consistent with the experimental values, with the high Pearson correlation coefficient values of 0.887 and 0.890 for Davis and KIBA, further confirming that the DualPG‐DTA model has good prediction accuracy. Kernel density estimation analysis (Figure 2C,D) shows that the distribution of predicted values from the DualPG‐DTA model and experimental values is highly consistent, proving that our model can accurately capture the overall distribution characteristics of the data.

#### 2.2.2 Ablation Experiments

As shown in Figure 1, DualPG‐DTA uses the node‐level features provided by the upstream pretrained models and the customized edge features to complete the construction of the molecular graph and protein graph, and then uses the GNN to extract graph‐level features and input them into the fusion prediction module to complete the output of the predictive results. To further investigate the effectiveness and necessity of these well‐designed modules in the DualPG‐DTA framework, a series of ablation studies were performed to decouple DualPG‐DTA networks. Concretely, we designed five variants of DualPG‐DTA as follows:

1. DualPG‐DTA *without molecular graph* (w/o MG): remove the part of molecular graph construction and GNN training (Figure 1A), and directly use the sequence‐level features (i.e., the average of all node‐level features) output by the Uni‐Mol pretrained model to input the fusion prediction module to complete the prediction.
2. DualPG‐DTA *without protein graph* (w/o PG): remove the part of protein graph construction and GNN training (Figure 1B), and directly use the sequence‐level features (i.e., the average of all node‐level features) output by the ESM‐2 pretrained model to input the fusion prediction module to complete the prediction.
3. DualPG‐DTA *without molecular graph and protein graph* (w/o MG/PG): remove both the parts of molecular graph and protein graph construction and the corresponding GNN training (Figure 1A,B).
4. DualPG‐DTA *without attention mechanism* (w/o AM): use GCN without attention mechanism to replace GATv2 (Figure 1A,B).
5. DualPG‐DTA *without dynamic attention mechanism* (w/o DAM): use GAT without dynamic attention mechanism to replace GATv2 in Figure 1A,B.

All these five variants of DualPG‐DTA were assessed on the Davis dataset, and the experimental setup and evaluation metrics are consistent with the original DualPG‐DTA for a fair comparison. As shown in Figure 2E–G, when the relevant part of molecular graph or protein graph is removed, the performance metrics (MSE, CI, and $r_{m}^{2}$) of the variant models (w/o MG and w/o PG) deteriorate to a certain extent. Meanwhile, when removing both the relevant parts of molecular graph and protein graph, the performance of the variant model (w/o MG/PG) will further decrease. These results demonstrate the necessity of the graph construction strategy in the DualPG‐DTA architecture for drug‐target binding affinity prediction tasks.

As shown in Figure 2H–J, when the GNN is replaced from GATv2 to GAT, the performance of the variant model (w/o DAM) decreases slightly, proving that the static linear transformation mechanism of GAT to calculate the attention coefficient is defective compared to the dynamic adjustment algorithm of GATv2. Meanwhile, when the GNN is replaced with GCN, the performance of the variant model (w/o AM) will drop significantly. One possible reason is that when a node updates its feature vector in GCN, it aggregates the feature information of neighboring nodes with equal weights, assuming that all neighboring nodes have the same influence on the current node, thereby ignoring that the importance of neighboring nodes may be different. Meanwhile, the weight assignment of GCN is fixed, usually based on the topological structure of the graph, while GAT automatically learns how to assign weights to each neighbor through training data. Collectively, these results indicate that GATv2 in the DualPG‐DTA architecture also plays a key role in drug‐target binding affinity prediction tasks.

#### 2.2.3 Y‐Scrambling Testing

Y‐Scrambling testing was employed to further evaluate the validity of the model. Specifically, a total of 10 random seeds from 1 to 10 were used to shuffle the labels of each benchmark dataset for training, validation, and testing. Figure 2K,L illustrates the differences between the test results after 10 Y‐Scrambling operations and the original data. Obviously, the original DualPG‐DTA model without Y‐Scrambling (blue sphere) significantly outperforms the 10 models after Y‐Scrambling (red sphere) in all evaluation indicators. These results indicate that the DualPG‐DTA model is statistically effective and can correctly capture the real and meaningful relationship between input variables and output variables.

### 2.3 Identifying CDK9 Inhibitors Featuring a Novel Scaffold via the DualPG‐DTA Model

**Figure 3:**
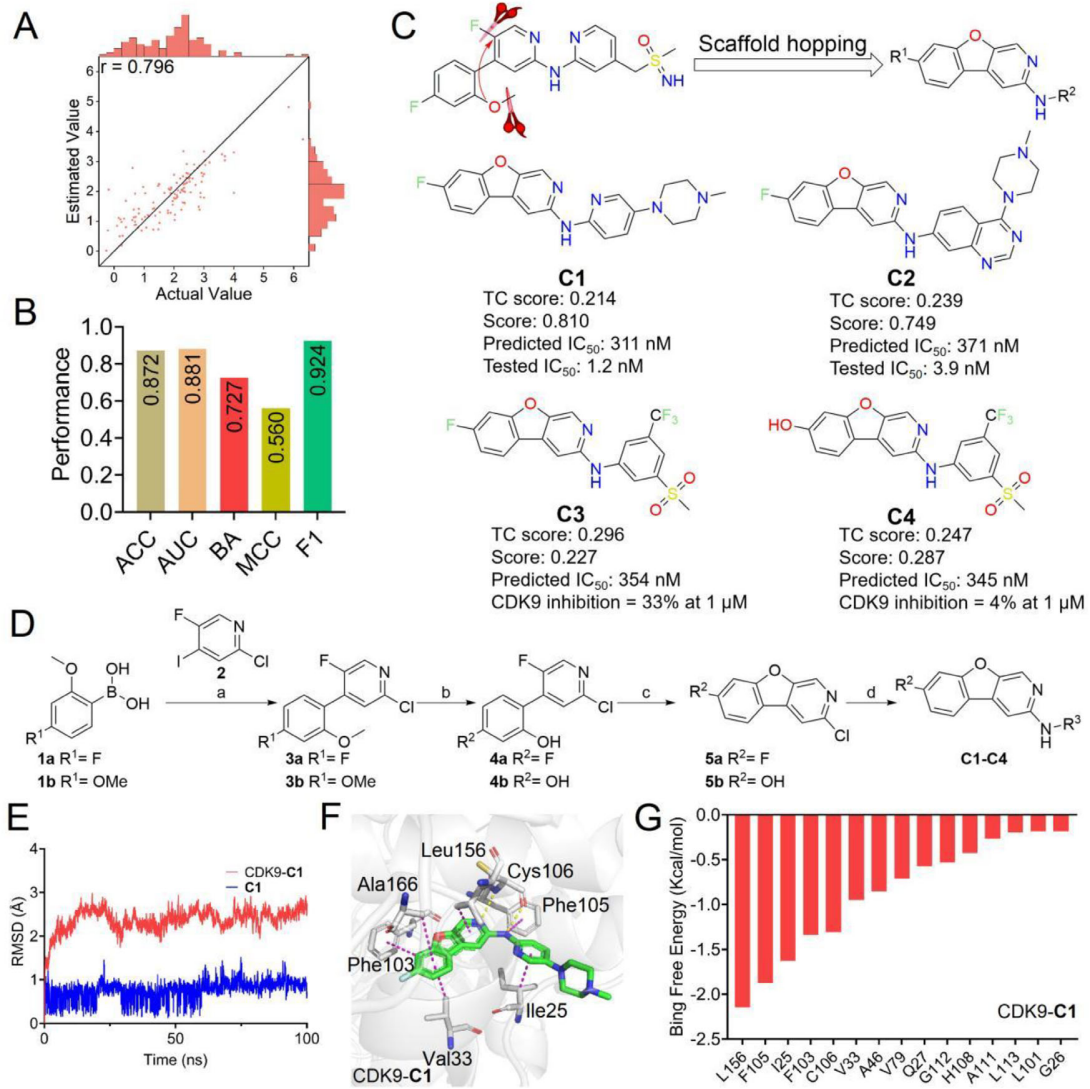
Identifying novel CDK9 inhibitors using the DualPG‐DTA model. A) Performance of DualPG‐DTA regression model on CDK9 inhibitor dataset. B) Performance of DualPG‐DTA classification model on CDK9 inhibitor dataset. C) Structural novelty scores, predictive scores, and experimental values of designed compounds **C1**–**C4**. Data are the mean of n ≥ 2. D) Synthetic routes for compounds **C1**–**C4**. E) RMSD plots for CDK9‐**C1** complex and compound **C1** during 100 ns MD simulations. F) Schematic representation of the protein‐ligand interactions between CDK9 and **C1**. G) Per‐residue decomposition of binding free energy calculated using MMPBSA for CDK9‐**C1**.

The translational application of predictive models in real‐world drug discovery workflows holds paramount significance, particularly in accelerating the identification of bioactive compounds and/or lead compounds. Motivated by this imperative, we utilized the DualPG‐DTA framework to discover novel CDK9 inhibitors. Notably, CDK9 plays a key role in controlling cellular transcription and has emerged as an attractive target for the treatment of acute myeloid leukemia (AML).^[^ ^24^, ^25^ ^]^ To accomplish this, we employed a dataset of ≈1,330 CDK9 inhibitors from our recent study,^[^ ^25^ ^]^ which exhibits high structural diversity and broad chemical coverage, to develop a regression model using the DualPG‐DTA framework for predicting drug‐target binding affinity (Figure S1, Supporting Information). As shown in **Figure** **3**A, this model achieved considerable performance for binding affinity prediction of CDK9‐inhibitors, with the r value of 0.796 on the testing set. Given the potential data heterogeneity arising from diverse experimental sources in the literature‐sourced CDK9 inhibitors dataset,^[^ ^25^, ^26^ ^]^ we incorporated a Sigmoid function into the DualPG‐DTA output layer to build a predictive classification model to address data variability. As shown in Table S2 (Supporting Information), the DualPG‐DTA model achieved the best performance across all evaluation metrics, with the highest ACC, AUC, BA, MCC, and F1 values of 0.872, 0.881, 0.727, 0.560, and 0.924 (Figure 3B), further demonstrating the powerful modeling and prediction capabilities of DualPG‐DTA.

While predictive models have demonstrated considerable utility in drug discovery, a persistent limitation lies in their reduced accuracy when evaluating compounds with novel structural scaffolds, particularly in scaffold hopping applications. Scaffold hopping enables circumvention of existing patent protections on drug/lead compound cores while generating new chemical entities for subsequent development. Analysis of the BAY1251152‐CDK9 docking pose (Figure S2, Supporting Information) confirmed the two critical hinge‐region hydrogen bonds via its aminopyridine group and Cys106 but also revealed that the sulfonimidamide group was solvent‐exposed. To exploit the adjacent hydrophobic cavity (Phe103, Val33, Lys48) reported in the literature to enhance binding, we implemented a scaffold‐hopping strategy through cyclization. Connecting the methoxy and fluorine substituents was hypothesized to improve rigidity and position the newly formed five‐membered ring into this cavity. Building upon this paradigm, four novel compounds (**C1**–**C4**, Figure 3C) were designed through scaffold‐hopping modifications of the established CDK9 inhibitor BAY1251152. Molecular similarity analysis using ECFP_4 fingerprints showed that these compounds are structurally novel, with lower Tanimoto coefficient scores of 0.214, 0.239, 0.296, and 0.247 (Figure 3C) relative to the parent compound.

We subsequently employed our established binding affinity regression and activity classification models to evaluate four novel compounds (**C1**–**C4**) for CDK9 inhibition. The regression model yielded moderate binding affinity predictions across all compounds, with predictive IC_50_ values ranging between 300–400 nM (Figure 3C). The classification model predictive results showed that the compounds **C1** and **C2** exhibited potential inhibitory activity against CDK9, with the high predictive scores of 0.810 and 0.749 (Figure 3C), while **C3** and **C4** showed limited inhibitory potential due to their lower predicted scores (0.227 and 0.287). Based on these results, we synthesized the compounds **C1**–**C4** (Figure 3D) and validated their inhibitory activity through biochemical assays. In vitro kinase assays results showed that **C1** and **C2** exhibited superior inhibitory activity against CDK9, with IC_50_ values of 1.2 and 3.9 nM, respectively (Figure 3C). In contrast, **C3** and **C4** showed weak CDK9 inhibitory activity, with inhibition rates of 33% and 4% at 1 µM, respectively. These experimental outcomes confirm the predictive capability of our model in identifying novel scaffolds, as evidenced by the discovery of two highly active and structurally distinct CDK9 inhibitors, **C1** and **C2**. Importantly, although **C3** and **C4** share the same dibenzo five‐membered heterocycle, their side‐chain modifications led to significantly reduced activity—a distinction accurately predicted by our model. This result highlights the model's sensitivity to subtle structural variations, even within the same scaffold family, and its ability to prioritize compounds with promising biological activity.

### 2.4 C1 Binds within CDK9 and Exhibits Favorable Selectivity Across the CDK Kinase Family

To evaluate the molecular mechanisms underlying the interaction between compound **C1** and CDK9, molecular docking and molecular dynamics simulations were performed. As shown in Figure 3E, the CDK9‐**C1** complex reached equilibrium after 20 ns, with the RMSD value stabilizing ≈ 2.5 Å. Binding free energy calculations via the Molecular Mechanics/Poisson‐Boltzmann Surface Area (MMPBSA) method estimated a ΔG value of −33.88 kcal mol^−1^ (Table S3, Supporting Information), suggesting a strong binding affinity, which aligns with the enzyme inhibition assay results. The most stable conformation of the CDK9‐**C1** complex (Figure 3F), as determined by principal component analysis (Figure S3A, Supporting Information), revealed that compound **C1** forms two hydrogen bonds with Cys106 in the hinge region of CDK9, which is crucial for its inhibitory activity. MD simulations demonstrated that the hydrogen bonds between **C1** and Cys106 remained stable throughout the simulation (Figure S3B; Table S4, Supporting Information). Additionally, five π‐H interactions were observed between **C1** and Leu156, Phe105, Ile25, Phe103, and Ala166. Free energy decomposition analysis identified Leu156, Phe105, Ile25, Phe103, Cys106, and Val33 as key residues that significantly contribute to the binding free energy, highlighting their essential roles in stabilizing the CDK9‐**C1** interactions (Figure 3G).

The kinase selectivity profile was systematically evaluated across the CDK family (**Table** **2**). Notably, **C1** exhibited potent inhibitory activity exclusively against CDK6, CDK9, and CDK16 (>90% inhibition at 200 nM), while showing limited inhibitory activity against other CDK isoforms (<75% inhibition at 200 nM). In general, compound **C1** exhibited high kinase selectivity for CDK9 and acts as a selective inhibitor of CDK9.

**Table 2:** Selectivity of compound **C1** against the CDK family.

Kinase	Inhibition rate^a)^ @200 nM	Kinase	Inhibition rate^a)^ @200 nM
CDK1/CycA2	2.37%	CDK12/CycK	21.25%
CDK2/CycE1	31.54%	CDK13/CycK	20.91%
CDK3/CycE1	20.68%	CDK14/CycY	50.66%
CDK4/CycD1	74.95%	CDK15/CycY	72.77%
CDK5/p35NCK	31.90%	CDK16/CycY	92.50%
CDK6/CycD1	95.55%	CDK17/CycY	60.79%
CDK7/CCNH/MNAT1	35.36%	CDK18/CycY	65.69%
CDK8/CycC	−2.01%	CDK19/CycC	−1.08%
CDK9/CycT1	91.50%	‐	‐

^a)^ The mean inhibition rate of **C1** was measured in duplicate at two independent times.

### 2.5 Inhibitory Efficacy and Mechanism Insights of C1 on Wild‐Type and Venetoclax Resistant AML Cells

**Figure 4:**
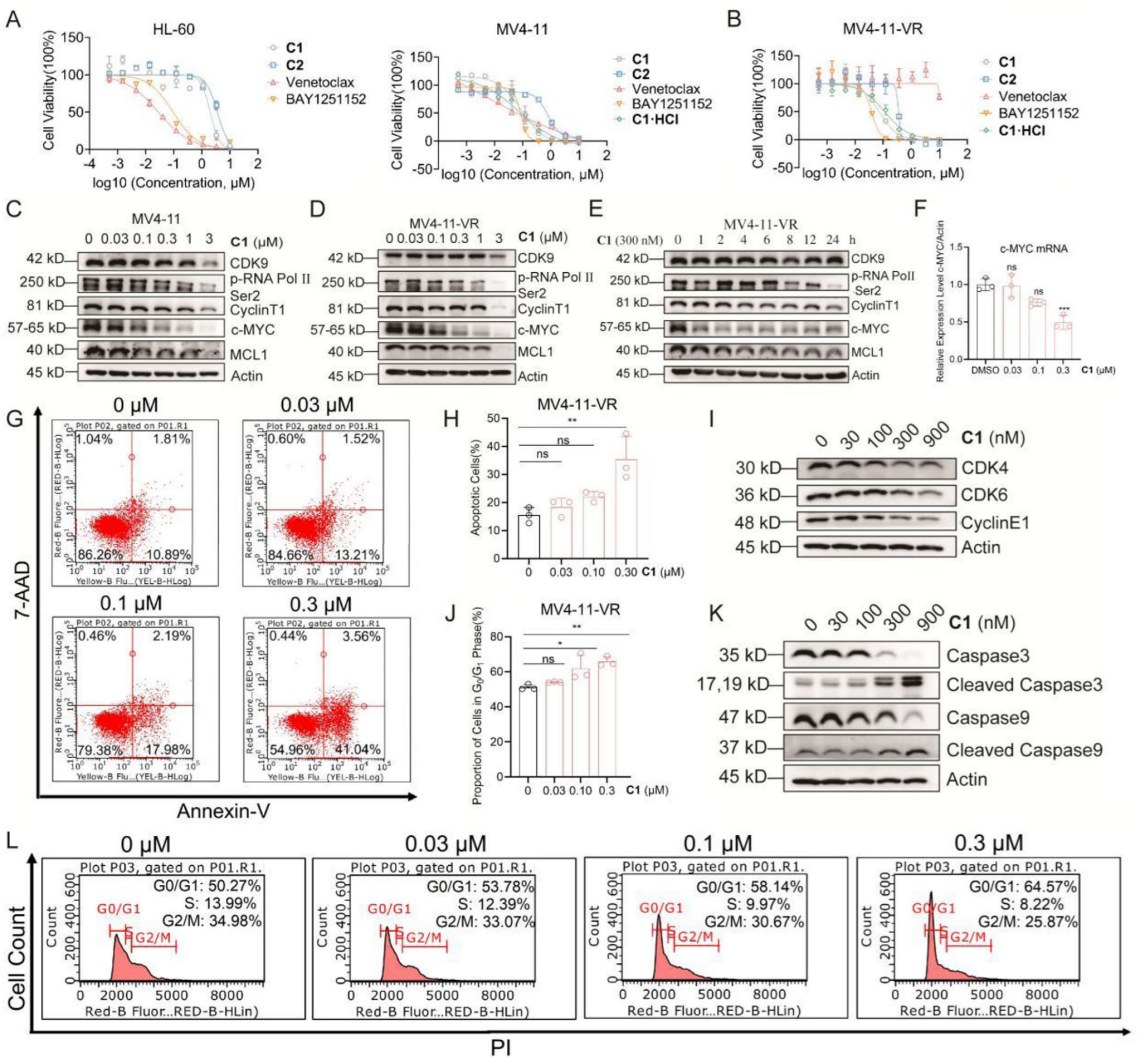
Inhibitory Activity and Mechanism of Compound **C1** on Acute Myeloid Leukemia Cells. A) Effects of **C1**, **C2**, Venetoclax, BAY1251152, and **C1·HCl** on cell viability in HL‐60 and MV4‐11 cell lines after 72 h treatment. B) Effects of **C1**, **C2**, Venetoclax, BAY1251152, and **C1·HCl** on cell viability in Venetoclax‐resistant MV4‐11 cell line (MV4‐11‐VR) after 72 h treatment. C) Effects of compound **C1** on CDK9‐driven signaling pathways in MV4‐11 and MV4‐11‐VR cells after 12 h treatment; D) Effects of compound **C1** on CDK9‐driven signaling pathways in MV4‐11‐VR cells after 12 h treatment; E) Effects of compound **C1** (300 nM) on CDK9‐driven signaling pathways in MV4‐11‐VR cells under different time conditions; F) qPCR analysis of c‐Myc mRNA in MV4‐11‐VR cells after 12 h compound **C1** treatment. Effects of compound **C1** on apoptosis G and H) and apoptosis‐related proteins I) in MV4‐11‐VR cells after 48 h treatment. Effects of compound **C1** on cell cycle (L, J) and cell cycle‐related proteins (K) in MV4‐11‐VR cells after 24 h treatment. Statistics, significance: one‐way ANOVA with Bonferroni correction (A, B, D); ns, not significant; ^*^ *p* < 0.05; ^**^ *p* < 0.01.

Compounds **C1** and **C2** were further evaluated for their anticancer activity on AML cell lines. As shown in **Figure** **4**A, **C1** showed favorable inhibitory effects on leukemia cell lines including HL‐60 and MV4‐11, with IC_50_ values of 2.47 ± 0.52 and 0.13 ± 0.03 µM, respectively. Meanwhile, **C1** exhibited a notable 24‐fold selectivity for the MV4‐11 cell line over the normal MRC‐5 cells, with an IC_50_ of 3.21 ± 0.15 µM for the latter. In comparison, the clinical‐stage CDK9 inhibitor BAY1251152 showed comparable potency against MV4‐11 (IC_50_ = 0.061 ± 0.01 µM) and MRC‐5 (IC_50_ = 0.03 ± 0.01 µM) cell lines, suggesting that compound **C1** may offer improved safety profiles (Figure S4A, Supporting Information).

Western blot (WB) was performed on MV4‐11 cells to elucidate the potential anticancer molecular mechanism of compound **C1**. As shown in Figure 4B, the phosphorylation level of RNAP II CTD (Ser2) was inhibited with **C1** treatment in a concentration‐dependent manner, indicating that **C1** could inhibit the activity of CDK9 in MV4‐11 cells. Annexin V‐FITC and PI staining were then conducted to verify whether **C1** affects cell proliferation through apoptosis. Figure S4C (Supporting Information) shows that when MV4‐11 cells were treated with **C1** at concentrations of 0.03, 0.1 and 0.3 µM for 48 h, the total number of early and late apoptotic cells was 12.32%, 16.93%, 20.3%, respectively. These results confirmed that **C1** induced cell apoptosis in a dose‐dependent manner, thereby contributing to its anticancer effect on MV4‐11 cells. Additionally, **C1** resulted in a notable concentration‐dependent G0/G1 phase cell cycle arrest in MV4‐11 cells, with the proportion of cells in the G0/G1 phase rising from 58.99% in the control group to 59.71%, 70.00% and 83.33% at 0.03, 0.1 and 0.3 µM of **C1**, respectively (Figure S4D, Supporting Information).

Our findings indicated that **C1** markedly reduces the expression levels of anti‐apoptotic proteins, including Mcl‐1 and c‐MYC, in MV4‐11 cells. Additionally, CDK9 inhibitors have been reported to regulate the expression of pro‐apoptotic protein BIM and induce sustained downregulation of Bcl‐2 mRNA transcription.^[^ ^27^ ^]^ Increased Mcl‐1 protein levels caused by compensatory upregulation, enhanced stability, or modified functionality and its binding to BIM after Venetoclax (a Bcl‐2 inhibitor) treatment in Venetoclax‐resistant AML cell lines and primary patient samples represent an intrinsic mechanism of resistance to Venetoclax in AML.^[^ ^28^ ^]^ Notably, ≈30% to 40% of AML patients are resistant to Venetoclax‐based regimens with relapse driven by drug resistance presenting a significant threat to survival.^[^ ^29^ ^]^ Downregulation of Mcl‐1 may indirectly overcome this intrinsic resistance mechanism, thereby enhancing the therapeutic efficacy of Venetoclax in AML. CDK9 inhibitors exhibit synergistic sensitization when combined with Bcl‐2 inhibitor Venetoclax in both in vitro and in vivo models of Venetoclax‐resistant AML.^[^ ^28^, ^30^ ^]^ Considering these factors, we tested the effects of compound **C1** on the Venetoclax‐resistant MV4‐11 (MV4‐11‐VR) cell line, which showed superior anti‐proliferative activity with an IC_50_ value of 0.063 ± 0.01 µM. Furthermore, in MV4‐11‐VR cells, **C1** led to a dose‐dependent downregulation of p‐RNAP II CTD (Ser2), Cyclin T1, c‐MYC, and Mcl‐1 (Figure 4D). Downregulation of c‐MYC protein was clearly observed as early as 1 h after the treatment of **C1** (0.3 µM), whereas the reduction in Mcl‐1 levels became evident at 2 h, slightly lagging behind the effects on c‐MYC (Figure 4E). Figure 4E indicated that the mRNA expression level of c‐MYC was decreased in a dose‐dependent manner with the treatment of **C1** ranging from 0.03 to 3 µM. Similar to its activity in MV4‐11 cells, **C1** also promoted apoptosis (Figure 4G,H) and induced G0/G1 phase cell cycle arrest in MV4‐11‐VR cells (Figure 4L,J), further supporting the potential efficacy of **C1** in overcoming Venetoclax resistance by inhibiting CDK9. In addition, **C1** mediated the activation of apoptosis‐associated proteins (e.g., cleaved Caspase‐3 and cleaved Caspase‐9, Figure 4K) and the downregulation of the phase‐associated proteins (e.g., CDK4/6 and Cyclin E1, Figure 4I).

### 2.6 Metabolic Properties of Compound C1

We further evaluated the in vitro metabolic stability of **C1** in human liver microsomes and in vivo pharmacokinetic (PK) properties via intravenous (i.v.) and oral (p.o.) administration. In human liver microsomes (Figure S4E, Supporting Information), **C1** exhibited high metabolic stability, with a T_1/2_ of 98.18 min and slow intrinsic clearance (CL_int_ 14.12 µL min^−1^ mg^−1^ protein), highlighting its potential as a promising candidate for further in vivo studies. Following i.v. (5 mg kg^−1^), **C1** had AUC_last_ and AUC_inf_ values of 412 and 489 h^*^ng mL^−1^, with a C_max_ of 229 ng mL^−1^ (**Table** **3**). P.o. (20 mg kg^−1^) of **C1** provided acceptable in vivo exposure, with AUC_last_ and AUC_inf_ values of 400 and 582 h^*^ng mL^−1^ and satisfactory oral bioavailability (F = 24.3%). Both i.v. and p.o. administration routes exhibited a long half‐life, with T_1/2_ values of 10.9 h and 13.5 h, respectively. These favorable PK properties of **C1** strongly support the feasibility of oral dosing for the further evaluation of its antitumor activity in vivo.

**Table 3:** Pharmacokinetic parameters of compound **C1** in Sprague Dawley rat.

Dose/routes	5 mg kg^−1^ [i.v.]	20 mg kg^−1^ [p.o.]
T_1/2_ (h)	10.9 ± 0.8	13.5 ± 5.8
T_max_ (h)	/	8.00 ± 0.8
C_max_ (ng/mL)	229.0 ± 20.3	25.5 ± 4.8
AUC_last_ (h^*^ng/mL)	412.0 ± 25.2	400.0 ± 60.4
AUC_inf_ (h^*^ng/mL)	489.0 ± 25.9	582.0 ± 129.4
CL (mL/min/kg)	171.0 ± 8.8	/
F (%)	/	24.3 ± 3.7

T_1/2_, terminal half‐life; T_max_, the time take to reach C_max_; C_max_, maximum drug concentration; AUC_last_, area under the curve between 0 and 24 h; AUC_inf_, area under the curve to infinity; CL, plasma clearance rate; F, oral bioavail ability. n = 3.

### 2.7 In Vivo Anticancer Activity of C1 in Xenograft Mouse Model

**Figure 5:**
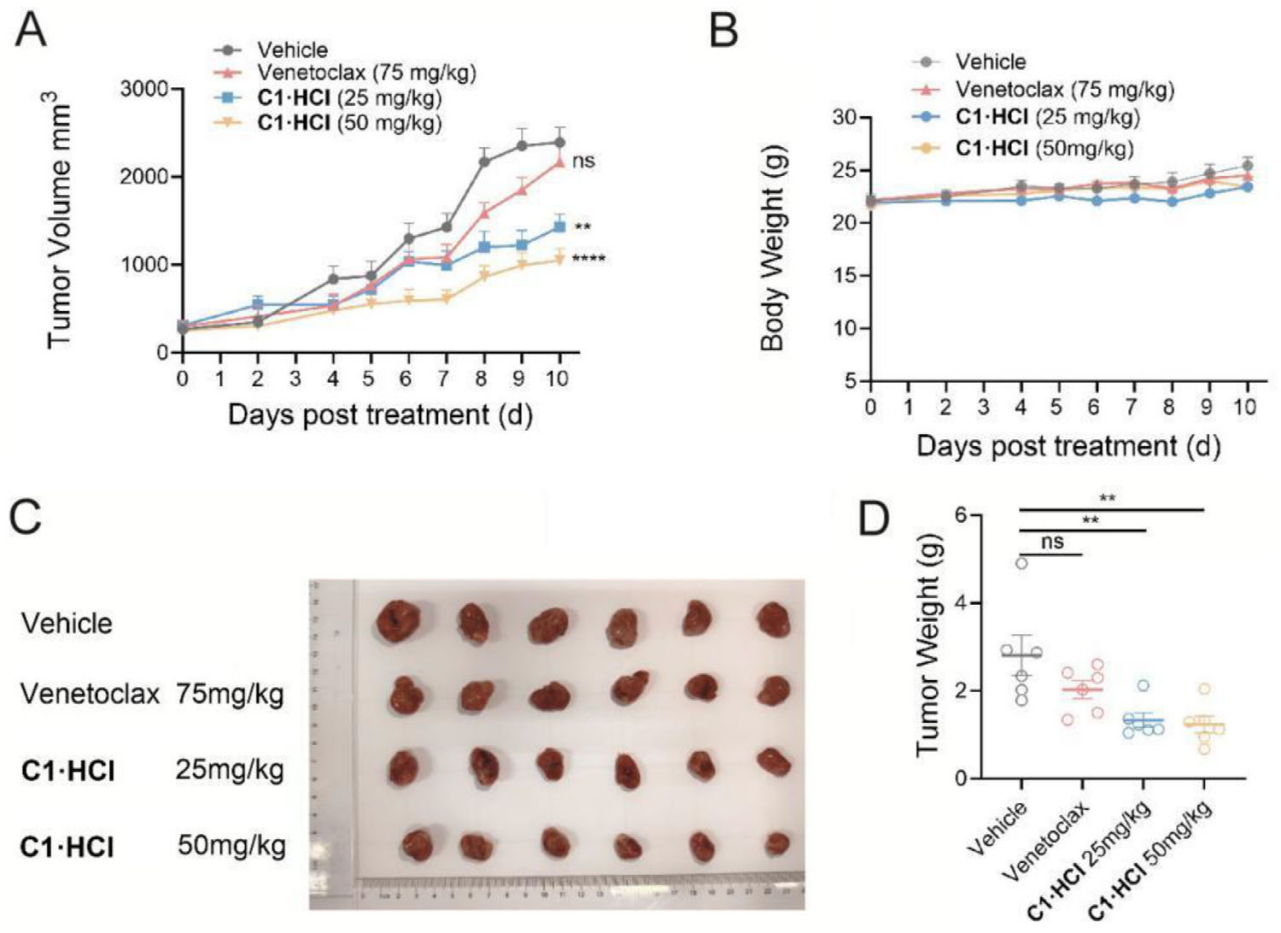
Tumorigenicity study on MV4‐11‐VR xenograft‐bearing mice. Tumor volume (A) and body weight (B) of MV4‐11‐VR xenograft‐bearing mice treated with **C1·HCl**. Final tumor size (C) and final tumor weight (D) after treatment for 10 days. Statistics, significance: one‐way ANOVA with Bonferroni correction (A, B, D); ns, not significant; ^*^ *p* < 0.05; ^**^ *p* < 0.01; ^***^ *p* < 0.001.

Given the favorable bioavailability (F═ 24.3 ± 3.7%) and T_1/2_ of 13.5 ± 5.8 h via p.o. administration, we aimed to evaluate the in vivo efficacy through daily oral administration. The MV4‐11‐VR xenograft‐bearing mice were treated daily for 11 days with **C1·HCl** (the hydrochloride salt of **C1**. The antiproliferative activity and WB analysis of compound **C1·HCl** against AML cell lines are presented in Figure 4A; Figure S4G, Supporting Information) administered orally at doses of 25 and 50 mg kg^−1^. Venetoclax (75 mg kg^−1^, p.o.) was included as a positive control. As illustrated in **Figure** **5**A, **C1·HCl** demonstrated superior tumor suppression compared to Venetoclax, with tumor growth inhibition (TGI) values of 47.2% and 62.7% for 25 and 50 mg kg^−1^, respectively, whereas Venetoclax (75 mg kg^−1^) did not exhibit significant inhibitory effects on tumor growth (Figure 5C and Figure 5D). Importantly, no significant weight loss was observed in any treatment group during the study (Figure 5B), underscoring the favorable tolerability of **C1·HCl**. These results highlight the potential of **C1** as a potent and well‐tolerated antitumor agent.

## 3 Conclusion

In the present study, we introduce DualPG‐DTA, an innovative DL framework that combines two pretrained large language models with GNNs with a multi‐head attention mechanism for predicting drug‐target binding affinity. Compared to eight existing ML and DL methods, systematic and comprehensive performance evaluation results show that DualPG‐DTA achieves the best predictive performance across all evaluation metrics on two commonly used benchmark datasets. Multi‐level validation testing, including visualizing the relationship between predicted and experimental values, ablation experiments, and Y‐Scrambling testing, further proves the rationality and effectiveness of the DualPG‐DTA architecture. Moreover, the scaffold hopping testing demonstrated the ability of the DualPG‐DTA model to accurately predict compounds with new scaffolds, which further led to the discovery of compound **C1** as a novel CDK9 inhibitor. In addition, **C1** showed good kinase spectrum selectivity, safety and PK properties, as well as anti‐AML activity in vitro and in vivo in Venetoclax‐resistant models. Compound **C1** offers potential for extending disease‐free survival and overall survival by overcoming Venetoclax resistance. Collectively, compound **C1** represents a promising lead compound for the development of therapies targeting AML, particularly for patients resistant to Venetoclax.

In addition, our DualPG‐DTA framework is designed to be general‐purpose and can, in principle, be applied to any target class, including GPCRs and ion channels, as long as both protein and molecule information are available. This broad applicability is supported by two key factors: (a) ESM‐2 embeddings capture universal sequence motifs, such as the seven‐transmembrane domains typical of GPCRs, and (b) the distance‐based protein graph representation adapts to any protein fold. That said, actual performance may vary depending on the quantity and quality of the training data available for specific target classes, so model accuracy for particular applications such as GPCRs or ion channels should be evaluated on a case‐by‐case basis.

Although DualPG‐DTA demonstrates leading‐performance in binding affinity prediction and novel scaffold identification, the model has certain limitations that warrant consideration, including its reliance on pre‐computed 3D molecular conformations, which may constrain applications involving dynamic conformational sampling, as well as its higher computational resource requirements during training compared to simpler single‐modality approaches such as DeepDTA; these trade‐offs between accuracy and efficiency present meaningful opportunities for future optimization.

## 4 Experimental Section

### Datasets

Two benchmark datasets (Davis^[^ ^31^ ^]^ and KIBA^[^ ^32^ ^]^) were utilized to evaluate and validate model performance. The basic information of the datasets is summarized in Table S5 (Supporting Information). The Davis dataset includes a large collection of binding affinity data between kinase targets and small molecules. These data are obtained through precise experimental methods and recorded as *K*_d_ values as a quantitative indicator of drug‐target binding affinity. *K*_d_ is often used to describe the tendency of a protein‐ligand complex to dissociate into free protein and free ligand under dynamic equilibrium. Currently, the Davis dataset encompasses over 30 000 protein‐ligand pairs, providing a broad range of binding affinities and allowing researchers to test and evaluate the accuracy and robustness of predictive models across different affinity levels. Given the large range of the original *K*_d_ values, which is not suitable for training DL models, we followed the approach of He et al.^[^ ^14^ ^]^ and transformed *K*_d_ values into logarithmic form p*K*_d_.

The KIBA dataset integrates experimental data on drug‐target interactions from multiple sources, including *K*_d_, *K*_i_, and IC_50_ values. To standardize these data, the dataset defines a composite score, called the KIBA score, which aims to provide a continuous and consistent measure of binding affinity. The original KIBA dataset includes 467 proteins and 52,498 small molecules. Considering the need for sample balance in the benchmark dataset, the data filtering method of He et al.^[^ ^14^ ^]^ was used to retain data for 229 proteins and 2,111 small molecules, ensuring that each target had at least ten activity measurements.

### Model Architecture

We present DualPG‐DTA, a DL framework that utilizes dual pretrained large language models with GATv2 networks for predicting drug‐target binding affinity. The DualPG‐DTA architecture comprises three core components: drug feature extraction module, protein feature extraction module, and multimodal fusion prediction module. Detailed technical specifications of each component are described in subsequent sections.

### Drug Feature Extraction Module

The initial input of the drug feature extraction module is a 1D SMILES string sequence. For feature extraction, we utilize Uni‐Mol,^[^ ^33^ ^]^ a comprehensive 3D molecular representation learning framework developed by DP Technology, and an open‐source chemical software package RDKit (https://www.rdkit.org/). Uni‐Mol was selected for three primary reasons: a) It was pretrained on over 200 million 3D molecular conformations, providing more comprehensive spatial information compared to 2D‐based models such as ChemBERTa, which is essential for capturing binding interactions; b) It delivers fine‐grained, atomic‐level embeddings (512‐D), making it well‐suited for processing with graph neural networks (GNNs); c) It has demonstrated state‐of‐the‐art performance on benchmark molecular property prediction tasks. Accordingly, we initialize the Uni‐Mol model with pretrained weights, input the drug SMILES from the benchmark datasets, and obtain the atomic‐level representations for each compound. Simultaneously, RDKit is employed to convert SMILES strings into molecular objects, and in‐house scripts are used to batch‐extract atomic and bond information for each compound, which is then matched with the atomic‐level representations generated by Uni‐Mol.

Next, we construct a 2D molecular graph for each compound, where nodes represent atoms and node features are the 512‐D atomic‐level representations output by Uni‐Mol. Edge features are encoded as 6‐D one‐hot vectors representing i) bond type (single, double, triple, or aromatic), ii) conjugation, and iii) hybridization (Figure 1A). During processing of molecular graphs in the initial layer of a three‐layer GATv2 network, the attention mechanism is mathematically formulated as:

(1) $$\alpha_{ij}=\mathrm{softma}x_{j}\left(\frac{W^{1}\cdot \left[h_{i}∥h_{j}\right]+b^{1}}{\sqrt{d_{k}}}\right)$$

where h_*i*_ and h_*j*_ are the node feature vectors of atom *i* and atom *j* respectively after being transformed by a learnable weight matrix *W* ^1^ and added with a bias *b* ^1^, ∥ denotes concatenation, *d_k_* is a scaling factor (usually set to the dimension of the concatenated vector), and α_*ij*_ represents the attention coefficient from atom *j* to atom *i*.

In the message passing step of the GATv2 network, the updated node feature 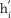 for atom *i* can be calculated as:

(2)

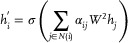

where *N*(*i*) is the set of neighboring atoms of atom *i*, *W* ^2^ is another learnable weight matrix, and σ is an activation function (such as ReLU).

These molecular graphs are fed into a three‐layer GATv2 network for training to produce graph‐level representations for each molecule, which serve as inputs to the subsequent fusion prediction module.

### Protein Feature Extraction Module

The protein feature extraction module processes 1D amino acid sequences using ESM‐2 and ESMFold^[^ ^34^ ^]^ as hierarchical feature extractors. ESM‐2 is a high‐precision protein sequence representation language model with 15 billion parameters. It leverages self‐supervised pretraining technology to learn evolutionary information and sequence features from extensive protein sequence databases. ESM‐2 generates high‐dimensional embeddings at the protein sequence and amino acid residue levels, which can be used for various downstream application tasks, including but not limited to sequence classification, functional prediction, and protein‐protein interaction prediction. Built upon ESM‐2, ESMFold is a protein structure prediction pretrained model that can capture complex patterns and long‐range interactions within protein sequences to achieve high‐precision structure predictions.

In the protein feature extraction module, we first utilize ESMFold to predict the 3D structure of each protein sequence in the benchmark dataset and output the prediction result as a PDB format file. Simultaneously, the ESM‐2 is used to extract residue‐level representations for each protein. Subsequently, we use an *in‐house* script to extract the 3D coordinates of each amino acid residue from the PDB file, compute the Euclidean distances between them, and generate a distance matrix. Finally, we construct a graph for each protein, where nodes represent amino acid residues and node features are determined by the 2560‐D atomic‐level representations output by ESM‐2. Edge weights are defined as the inverse Euclidean distances between Cα atoms, using a cutoff of 8Å. Similar to the drug feature extraction module, the protein graph is then input into a three‐layer GATv2 network for training and outputting graph‐level representations.

### Multimodal Fusion Prediction Module

The multimodal fusion prediction module orchestrates a hierarchical integration of drug and protein features through sequential transformation stages. After concatenating the drug and protein embeddings, the module employs a multi‐head attention mechanism to capture diverse interaction patterns across different feature subspaces. Specifically, the concatenated features are processed by a multi‐head self‐attention layer with 8 heads, where each head learns distinct attention weights:

(3) $$\mathrm{MultiHead}\left(Q,K,V\right)=\mathrm{Concat}\left(\mathrm{hea}d_{1},\text{…},\mathrm{hea}d_{8}\right)W^{O}$$

where each head is computed as:

(4) $$\mathrm{hea}d_{i}=\mathrm{Attention}\left(QW_{i}^{Q},KW_{i}^{K},VW_{i}^{V}\right)$$

This allows the model to jointly attend to information from different representation subspaces, enhancing its ability to capture complex drug‐target interactions. The output is then passed through fully connected layers (FCLs) with batch normalization and ReLU activation for dimensionality reduction and final affinity prediction.

The first FCL projects the concatenated features from 3072 to 1280 D, coupled with batch normalization (BN) and rectified linear unit (ReLU) activation to eliminate redundant cross‐modal correlations while retaining biochemically salient interaction signatures. This is followed by a second compression phase (1280 → 320 D) that further distills the feature space to isolate critical binding determinants, including hydrogen bonding patterns and hydrophobic complementarity. The final FCL employs linear activation to map the 320‐D refined features into a continuous binding affinity prediction. In the BN layers between these FCLs, the normalization process for each mini‐batch of data can be expressed as:

(5) $${\overset{⌢}{x}}_{i}=\frac{x_{i}-\mu_{B}}{\sigma_{B}^{2}+\in }\cdot \gamma +\beta$$

where x_i_ is the input feature, μ_B_ and $\sigma_{B}^{2}$ are the mean and variance of the current mini‐batch data respectively, ε is a small constant to avoid division by zero, and γ and β are learnable parameters for scaling and shifting the normalized data.

The BN layers normalize each mini‐batch of data to alleviate the issue of internal covariate shift, thereby accelerating network convergence and preventing gradient explosion or vanishing, even with relatively large learning rates. The ReLU layers introduce non‐linear activation functions to help the network capture the non‐linear relationships within the data and alleviate the vanishing gradient problem. Depending on the training dataset used, the prediction module outputs can include the *K*_d_, *K*_i_, half IC_50_, or other affinity scores between drugs and targets as determined by specific algorithms.

### Training Protocol, Hyperparameters Optimization, and Evaluation Metrics

Training a DL model is an iterative process where the model adaptively adjusts its parameters to progressively enhance prediction accuracy for a specific task. The ultimate goal of this process is to enable the model to perform well on unseen data, thereby demonstrating high generalization capability. In this study, a fivefold cross‐validation strategy was adopted^[^ ^3^ ^]^ to minimize the impact of random errors in the evaluation process and enhance the reliability of the assessment results.

During the model training process, we employed the Optuna open‐source library (https://github.com/optuna/optuna) to perform Bayesian optimization of the hyperparameters. The search space used in the optimization process and the final hyperparameters employed by the model are summarized in Table S6 (Supporting Information).

In this study, three commonly used metrics including MSE, CI, and $r_{m}^{2}$ metrics are used to evaluate the performance of the DualPG‐DTA model.

### In Vitro CDK9 Kinase Assay

CDK9 inhibition assay was conducted by Huawei Pharmaceutical Co., Ltd. (China) in accordance with standardized protocols.^[^ ^35^ ^]^ Enzymatic reactions were performed at 30 °C for 40 min in a 50 µL reaction mixture containing 40 mM Tris (pH 7.4), 10 mM MgCl_2_, 0.1 mg mL^−1^ BSA, 1 mM DTT, 10 µM ATP, 0.2 µg mL^−1^ CDK9 kinase, and 100 µM lipid substrate. Test compounds were prepared in 10% DMSO, and 5 µL of the diluted solution was added to the reaction, ensuring a final DMSO concentration of 1%. Kinase activity was evaluated using the Kinase‐Glo Plus luminescence assay kit, which determines the residual ATP concentration post‐reaction. The luminescent signal is directly proportional to the remaining ATP levels and inversely proportional to kinase activity. IC_50_ values were determined through nonlinear regression analysis with a normalized dose−response curve, using Prism GraphPad software.

### Molecular Docking

The X‐ray crystal structure of CDK9 (PDB ID: 4BCI) was retrieved from the Protein Data Bank and processed using the Protein Preparation Wizard in Maestro 12.8. The preparation steps included bond order assignment, hydrogen addition, and disulfide bond completion. The final protein structure was subjected to restrained minimization with a 0.30 Å RMSD cutoff, utilizing the OPLS‐2005 force field. The 3D conformation of the ligand was optimized using LigPrep in Schrödinger, also employing the OPLS‐2005 force field. The docking grid was generated via the Receptor Grid Generation tool, using the co‐crystallized ligand as the centroid to define the binding pocket. Molecular docking was performed in XP mode with default Glide settings. All structural figures were created using PyMOL (https://pymol.org/2/).

### Molecular Dynamics Simulation

Molecular charges were calculated using Gaussian HF/6‐31G^*^ with the RESP fitting method. The FF14SB force field was used for the protein, while GAFF was applied for the ligand. The system was solvated in a 12 Å TIP3P water box and neutralized with sodium and chloride ions. MD simulations were performed using the PMEMD.CUDA module of Amber 22 for 100 ns. The SHAKE algorithm was utilized to impose constraints on hydrogen bonds, while electrostatic interactions were computed employing the particle mesh Ewald method, using the particle mesh Ewald method with a 10 Å cutoff distance. Prior to simulation, the system underwent energy minimization to eliminate atomic clashes, employing 1000 steps each of steepest descent and conjugate gradient methods. Subsequent to minimization, the system underwent a gradual heating protocol under NVT ensemble conditions for 50 ps, regulated by a Langevin thermostat to ensure a controlled temperature increase from 0 to 310 K. This was followed by a 100 ps NPT equilibration phase, where the system was maintained at 310 K and 1 atm pressure for density stabilization and pressure coupling. An additional 100 ps equilibration was conducted to ensure thermal and pressure stability. After equilibration, a 100 ns production MD simulation was performed, with trajectory data recorded every 25 000 steps, yielding a total of 2000 frames for further analysis. Trajectory analysis was performed utilizing the CPPTRAJ module within Amber 22.

### Binding Free Energy Analysis

The MMPBSA methodology was utilized to determine the free energy of the final state based on molecular dynamics trajectory data, employing the gmx_MMPBSA program. The PB model was implemented with an ionic strength of 0.100 M to simulate the solution environment, utilizing default atomic radii settings. Additionally, free energy decomposition methods were applied to analyze the contribution of amino acids near the binding site to the overall binding free energy.

### Hydrogen Bond Analysis

We analyzed protein‐molecule complexes using the CPPTRAJ module within the Amber22 software suite. The 2000‐frame trajectory was aligned for further analysis, allowing us to quantify hydrogen bond lifetimes, occupancies, and average distances.

### Kinase Selectivity Assay

The assay buffer was prepared by combining 50 mM Hepes, 10 mM MgCl_2_, 0.01% Brij35, 1 mM EGTA, and 2 mM DTT in ddH_2_O. Two distinct 2× solutions were created using the assay buffer: one for ATP and substrate, and the other for kinase and metal. A volume of 40 nL of each test compound was transferred into a 384‐well plate utilizing the Echo 655 liquid handler. To each well, 2 µL of the 2× kinase and metal solution was added, followed by a 10‐min incubation at 25 °C. Next, 2 µL of the 2× ATP and substrate solution was added to each well, and the plate was incubated for 60 min at 25 °C. After this incubation, 4 µL of ADP‐Glo reagent was introduced, and the plate was incubated for an additional 40 min at 25 °C. Finally, 8 µL of kinase detection reagent was added to each well, and the plate was subjected to a further 40‐min incubation at 25 °C. Luminescence signals were subsequently recorded using a microplate reader.

### Cells and Reagents

MV4‐11 (RRID:CVCL_0064, acute myeloid leukemia, purchased from ATCC, Cat# CRL‐9591) and HL‐60 (RRID:CVCL_0002, promyelocytic leukemia, purchased from ATCC, Cat# CCL‐240) were obtained directly from ATCC (Manassas, VA, USA). Authentication of MV4‐11 and HL‐60 cell lines was performed by short tandem repeat (STR) profiling (Guangzhou IGEBIO Co., Ltd.), confirming no cross‐contamination with other human cell lines. MV4‐11 and MV4‐11‐VR cells were maintained in IMDM medium supplemented with 10% FBS, 100 U mL^−1^ penicillin, and 50 mg mL^−1^ streptomycin. MV4‐11‐VR cells required an additional 2 µM Venetoclax to maintain drug resistance during culture, and HL‐60 cells were maintained in 1640 medium. All the above cells were cultured at 37 °C in a humidified air incubator. Compounds were dissolved in 100% DMSO (Sigma‐Aldrich).

### Antiproliferation Cell Assay

Cells in the logarithmic growth phase were plated in 96‐well plates (15 000−20 000 cells per well) overnight. The compounds at a series of concentrations were added and cocultured for the further 72 h, and CCK8 (Dojindo Molecular Technologies Inc., Japan) was added into each well and coincubated for 2−4 h. The absorbance values of OD_450_ and OD_650_ were measured on an Envision multilabel Reader (Perkin Elmer). The cell viability rate (V%) of each well was calculated using equation: V (%) = (As − Ac) / (Ab − Ac) × 100. (A: OD_450_−OD_650_, s: sample, b: blank, c: control). IC_50_ for each compound was calculated using GraphPad Prism 9.5.1. Three duplications were set for each dose, and experiments were repeated for at least three times.

### Western Blot Analysis

Protein immunoblotting analysis was determined according to the method recommended by Cell Signaling Technology (CST, USA). Briefly, after 12 h of cell culture, cells were treated with the indicated compounds for the indicated time periods. Then, cells were incubated in 1× SDS loading buffer (62.5 mm Tris‐HCl, pH 6.8, 2% w/v SDS, 10% glycerol, 50 mm DTT, 0.01% w/v bromophenol blue, as recommended by CST) and cell lysates were boiled and centrifuged to load the supernatant onto an SDS‐PAGE gel, and electrophoresis was performed to separate the proteins. The separated proteins were then electrotransferred to a PVDF membrane. The PVDF membrane was incubated with primary antibody overnight at 4 °C (or for 2 h at room temperature) and then with secondary antibody for 2 h at room temperature. Protein bands were visualized using an ECL Western blotting detection kit (Thermo Scientific, Waltham, MA, USA) and chemiluminescent signals were detected with an Amersham Imager 600 system (GE, Boston, MA, USA) according to the manufacturer's instructions. Antibodies were purchased from Cell Signaling Technology (CST, Boston, MA, USA) as follows: anti‐CDK9 (#2316), anti‐p‐RNAP II CTD (Ser2) (#13499), GAPDH (#2118), Mcl‐1 (#4572), c‐MYC (# 18583), CyclinT1 (#D1B6G) enzyme‐linked anti‐rabbit IgG (#7074), enzyme‐linked anti‐mouse Ig (#7076). Some of the antibodies were purchased from Beyotime, such as Actin (AA128).

### qPCR Assay

Total RNA was extracted from 6‐well plates (5 × 106 cells per well) by first incubating and harvesting the test compounds together with the test compound or vector control (DMSO) for 12 h. RNA was extracted using Trizol reagent (ThermoFisher #15596018) and quantified using a Thermo/NanoD2000C Ultra‐Micro UV Spectrophotometer. Photometer for quantification. Reverse transcription was performed using the HiScript IV All‐in‐One Ultra RT SuperMix for qPCR (+gDNA wiper) kit for qPCR (Vazyme Biotech Co., ltd, Nanjing, China) according to the manufacturer's instructions. Subsequently, the reverse transcription products were amplified using SYBR® Green Pro Taq HS Premix for qPCR Kit II (#AG11702, China).

The primer sequences were as follows:

c‐MYCF_: CCTGGTGCTCCATGAGGAGAC;

c‐MYCR_: CAGACTCTGACCTTTTGCCAGG;

β‐Actin_F: 5′‐CACCATTGGGCAATGAGCGGTTC‐3′;

β‐Actin_R: 5′‐AGGTCTTTGCGGATGTCCACGT‐3′.

All reactions were performed in triplicate and relative gene expression levels were normalized to β‐actin using the 2‐ΔΔCt method. Melting curves were routinely performed to determine the specificity of the PCR.

### Apoptosis Assay

The cell apoptosis analysis was carried out according to the procedure provided by BD Bioscience (Fisher Scientific, USA) using a PE Annexin V Apoptosis Detection Kit (#559763, BD Bioscience). Briefly, cells were treated with the indicated compounds for 48 h. Then, cells were collected and washed twice with cold PBS. Cells (2 × 106) were resuspended in 1× BD binding buffer solution (#556454, BD Bioscience) and then stained with 7‐ADD (#559925, BD Bioscience) and Annexin V‐PE (#556422, BD) in the dark for 15 min. BD binding buffer solution (1×) was added to stop the staining. The cells were then analyzed using a Guava EasyCyte flow cytometer (Merck, USA).

### Cell Cycle Analysis

The Cell Cycle Analysis Kit (No. C1052; Beyotime, Shanghai, China) was used for cell cycle assays. Briefly, cells were treated with the indicated compounds for 24 h. Cells were then collected and washed twice with cold PBS, centrifuged to discard the supernatant, and fixed in 70% ethanol for 12 h at 4 °C and centrifuged at 1200 rpm to discard the supernatant. 0.5 mL of propidium iodide staining solution was added to each tube of cell samples, and the cell precipitates were slowly and fully resuspended in a warm bath at 37 °C away from light for 30 min. Subsequently, they can be stored at 4 °C or in an ice bath protected from light. A Guava EasyCyte flow cytometer (Merck, USA) was used to analyze the percentage of cells at different stages of the cell cycle. Each experiment was repeated three times.

### Liver Microsome Assay

Liver microsome assay was carried out by ICE Bioscience Inc. (China). Liver microsomes (LM) were prepared at a concentration of 0.5587 mg mL^−1^ in 100 mm PBS (pH 7.4). The incubation solution was made by adding 358 µL of the LM solution to each well. To this, 40 µL of either a 10 mM NADPH solution in PBS or 40 µL of PBS alone (for controls) was added. The plate was mixed briefly at 800 rpm for 10 s and incubated at 37 °C for 10 min. The reaction was initiated by adding 2 µL of a 10 µm test compound solution in PBS. At designated time points (0.5, 15, 30, 45, and 60 min), 50 µL of the incubation mixture was sampled and transferred to a new plate containing 200 µL of ice‐cold MeOH with IS. The plate was then centrifuged at 3220 × g for 40 min at 4 °C. Following centrifugation, 100 µL of the supernatant was transferred to a new plate and diluted with ultrapure water as required for LC‐MS/MS analysis.

### Pharmacokinetics Study

All animal experiments in this study were conducted in accordance with the guidelines approved by ICE Bioscience Inc. (China). Male Sprague Dawley rats (6–8 weeks old, weighing 200–300 g, SPF grade), sourced from Beijing Biotechnology Co., Ltd., were randomly assigned to two groups (n = 3 per group). One group received the test compound via i.v. injection, while the other group was dosed p.o. For the i.v. administration, the test compound was administered at a dose of 2 mg kg^−1^ in a 2.5 mg mL^−1^ solution composed of 10% DMSO, 15% Solutol HS‐15, and 75% saline. The oral group was given the test compound at a dose of 20 mg kg^−1^ in a 2 mg mL^−1^ suspension prepared using 0.5% CMC‐Na. Blood samples were collected at the following time points: 5, 15, 30 min, and 1, 2, 4, 6, 8, and 24 h post‐dosing. The blood was drawn and immediately transferred into plastic microcentrifuge tubes containing EDTA‐K_2_ anticoagulant. Samples were then centrifuged at 4,000 × g for 5 min at 4 °C. The supernatant was carefully transferred to fresh microcentrifuge tubes without anticoagulant and stored at −75 ± 15 °C until subsequent analysis.

### In Vivo Experiments

Female Balb/c nude mice (4‐5 weeks old) were purchased from Guangdong Pharmachem Biotechnology Co., Ltd. All animal experiments were conducted in accordance with the experimental protocol approved by the Ethics Committee of the Animal Experimentation Center at South China University of Technology. The animal experiment ethics approval number is AEC 2024090. Then 1 × 10^7^ MV4‐11‐VR cells were injected subcutaneously in the right axilla of SCID mice. When the average tumor volume reached 100–200 mm^3^, the mice were randomly grouped according to tumor volume (n = 6). The drug was dissolved in 5% DMSO, while 0.5% CMC Na aqueous solution was added. Tumor volume and body weight were monitored once a day. Tumor volume was calculated as L × W^2^/2, where L is the length of the tumor and W is the width of the tumor.

### Statistical Analysis and Data Availability

Statistical analyses were performed using GraphPad Prism 9.5.1 (GraphPad Software, Inc., La Jolla, CA, USA). Unpaired Student's *t*‐test was used to perform statistical analysis between the two groups. One‐Way ANOVA was used for comparisons among multiple groups. The level of significance was set at ^*^ *p* < 0.05, ^**^ *p* < 0.01, and ^***^ *p* < 0.001. The data generated during and/or analyzed in this study are available from the corresponding author upon reasonable request.

## Conflict of Interest

The authors declare no conflict of interest.

## Supporting information

Supporting Information

## Data Availability

All source code of DualPG‐DTA and data used in this work can be obtained from https://github.com/idruglab/DualPG‐DTA. The remaining data or questions regarding this study are available to the corresponding author upon request (Ling Wang: lingwang@scut.edu.cn).
