# Supplementary material for: DualPG‐DTA: A Large Language Model‐Powered Graph Neural Network Framework for Enhanced Drug‐Target Affinity Prediction and Discovery of Novel CDK9 Inhibitors Exhibiting In Vivo Anti‐Leukemia Activity
Source: Adv Sci (Weinh). 2026 Jan 27;13(12):e13099. doi: 10.1002/advs.202513099 (PMC12948262; doi:10.1002/advs.202513099)

**DualPG-DTA: A Large Language Model-Powered Graph Neural Network Framework for Enhanced Drug-Target Affinity Prediction and Discovery of Novel CDK9 Inhibitors Exhibiting In Vivo Anti-Leukemia Activity**

Yihao Chen^1,5^, Jindi Huang^1,5^, Cong Liu^2,3,5^, Shipeng Zhang^1^, Xinze Li^1^, Zhang Zhang^2,3,^*, Tie-Gen Chen^4,^* and Ling Wang^1,^*

*^1^Joint International Research Laboratory of Synthetic Biology and Medicine, Ministry of Education, Guangdong Provincial Key Laboratory of Fermentation and Enzyme Engineering, Guangdong Provincial Engineering and Technology Research Center of Biopharmaceuticals, School of Biology and Biological Engineering, South China University of Technology, Guangzhou 510006, China.*

*^2^State Key Laboratory of Bioactive Molecules and Druggability Assessment, School of Pharmacy, Jinan University, Guangzhou, 510632, China.*

*^3^International Cooperative Laboratory of Traditional Chinese Medicine Modernization and Innovative Drug Discovery of Chinese Ministry of Education, Guangzhou City Key Laboratory of Precision Chemical Drug Development, School of Pharmacy, Jinan University, Guangzhou, 510632, China.*

*^4^Zhongshan Institute for Drug Discovery, Shanghai Institute of Materia Medica, Chinese Academy of Sciences, Zhongshan, 528400, China; Shanghai Institute of Materia Medica, Chinese Academy of Sciences, Shanghai, 201203, China.*

*^5^These authors contributed equally.*

**Corresponding author. zhang_zhang@jnu.edu.cn (Z. Zhang)*[*, chentiegen@simm.ac.cn*](mailto:，chentiegen@simm.ac.cn) *(T. Chen),* [*lingwang@scut.edu.cn*](mailto:lingwang@scut.edu.cn) *(L. Wang).*

**
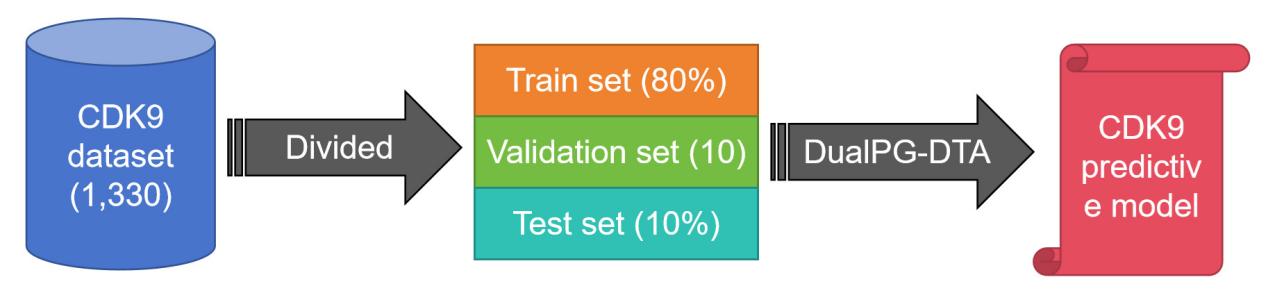
**

**Supplementary Figure 1.** The detailed workflow for constructing the CDK9 model.

**Supplementary Table 1.** Performance results of DualPG-DTA model on PDBBind v2020 refined set.

| Method | MSE | MAE | PCC |
| --- | --- | --- | --- |
| StructureNet (2025) | 1.964 | 1.125 | 0.683 |
| PLAIG (2025) | 1.564 | 0.997 | 0.763 |
| GNNSeq (2025) | 1.510 | 0.957 | 0.784 |
| DualPG-DTA (Our) | 1.384 | 0.931 | 0.776 |

**Supplementary Table 2.** Performance results of DualPG-DTA and existing models on CDK9 inhibitor dataset.

| Drug Representation | Method | ACC | AUC | BA | MCC | F1 |
| --- | --- | --- | --- | --- | --- | --- |
| PharmacoPFP | SVM | 0.789 | 0.747 | 0.530 | 0.089 | 0.879 |
|  | XGBoost | 0.805 | 0.823 | 0.588 | 0.221 | 0.886 |
| Morgan | SVM | 0.827 | 0.763 | 0.635 | 0.327 | 0.899 |
|  | XGBoost | 0.835 | 0.848 | 0.672 | 0.386 | 0.902 |
| AtomPairs | SVM | 0.835 | 0.870 | 0.655 | 0.367 | 0.903 |
|  | XGBoost | 0.842 | 0.878 | 0.660 | 0.389 | 0.907 |
| Graph | GCN | 0.835 | 0.875 | 0.672 | 0.386 | 0.902 |
|  | GAT | 0.835 | 0.812 | 0.558 | 0.261 | 0.908 |
|  | Attentive FP | 0.827 | 0.843 | 0.570 | 0.240 | 0.902 |
|  | FP-GNN | 0.850 | 0.830 | 0.681 | 0.427 | 0.912 |
|  | DualPG-DTA | **0.872** | **0.881** | **0.727** | **0.560** | **0.924** |

**
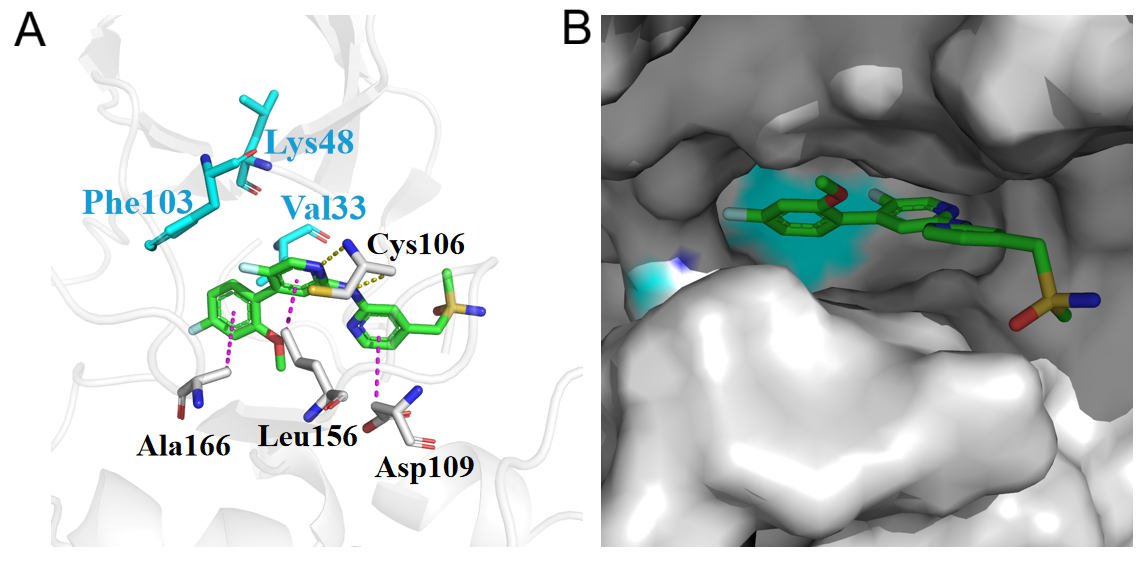
**

**Supplementary Figure 2.** Predicted binding mode (a) and surface representation (b) of BAY1251152 and CDK9 (PDB ID: 4BCI). Hydrogen bonds are represented by yellow dashed lines. π-stacking interactions are indicated by magenta dashed lines. Molecular docking was carried out with the Glide docking. All figures were generated using PyMOL (https://pymol.org/2/).

**Supplementary Table 3.** Binding free energy results based on MMPBSA method.

| Systems | CDK9-**C1** (kcal/mol) |
| --- | --- |
| ΔE_vdw_ | -48.15 ± 3.50 |
| ΔE_ele_ | -17.34 ± 3.11 |
| ΔG_gas_ | -65.49 ± 4.91 |
| ΔG_solv_ | 31.61 ± 3.10 |
| ΔG_total_ | -33.88 ± 4.22 |

**
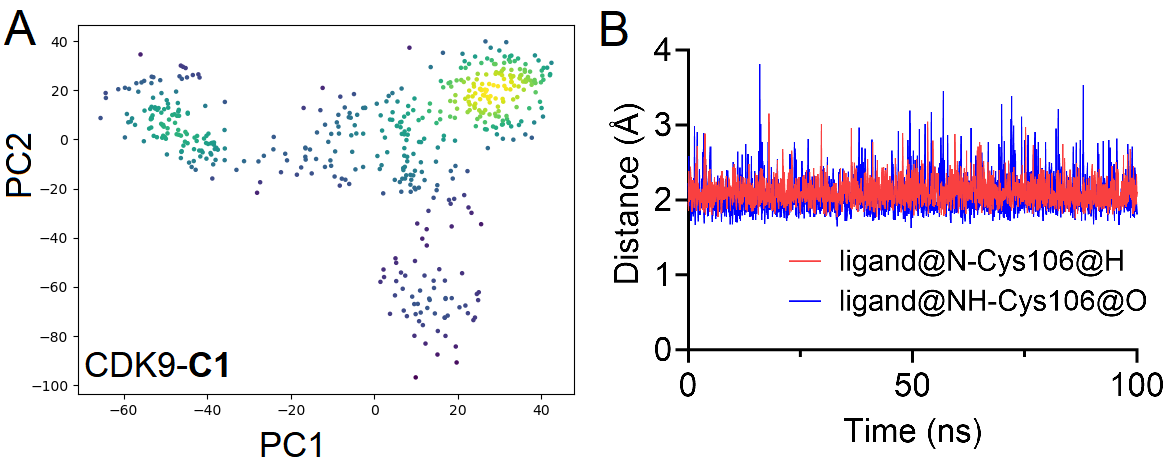
**

**Supplementary Figure 3.** Molecular dynamics simulations of **C1** with CDK9. (A) principal component analysis of CDK9-**C1** complex. (B) Distance analysis of hydrogen bonds with Cys106.

**Supplementary Table 4.** Hydrogen bond with Cys106 analysis from the results of MD simulations.

| Hydrogen bond ID | ligand@N-Cys106@H | ligand@NH-Cys106@O |
| --- | --- | --- |
| Acceptor | ligand@N | Cys106@O |
| Donor | Cys106@N-H | ligand@N-H |
| Occupancy (%) | 39.50% | 56.90% |
| Avg distance (Å) | 2.92 | 2.87 |
| Occurrences | 471 | 460 |
| Avg lifetime (frames) | 1.677 | 2.474 |
| Total lifetime (frames) | 790 | 1138 |

Note: The total number of simulation frames was 2000.

**
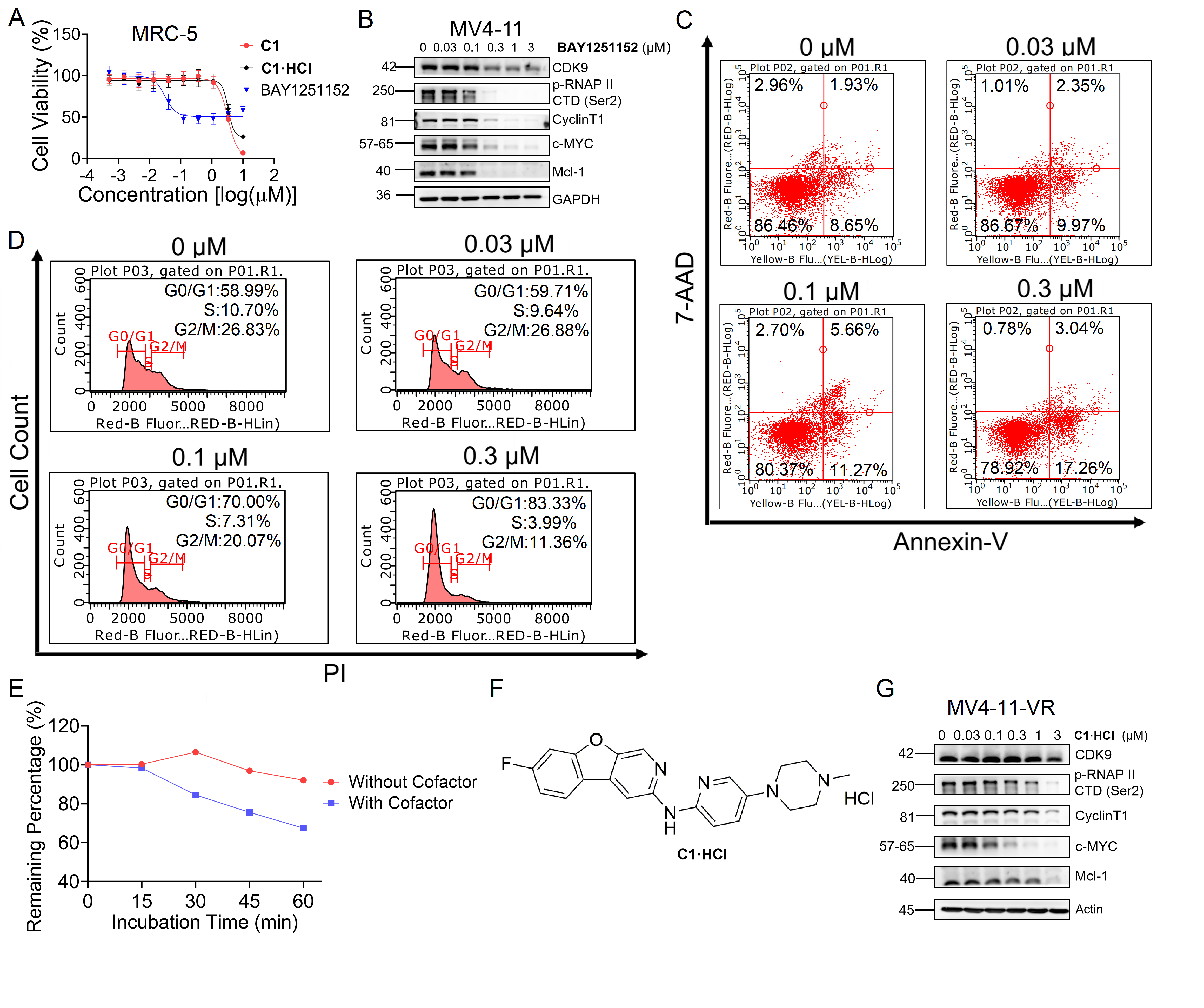
**

**Supplementary Figure 4.** Biological activity study of compound **C1** and its hydrochloride salt (**C1·HCl**). (A) The cell viability of **C1** in MRC-5 cells. (B) Effect of BAY1251152 on CDK9-driven signaling pathways in MV4-11 cells after 12 h treatment. (C) Effect of **C1** in MV4-11 cell apoptosis after 48 h treatment. (D) Effect of **C1** on cell cycle in MV4-11 cells after 24 h treatment. (E) Metabolic stability of **C1** in human liver microsomes. (F) The structure of **C1·HCl**. (G) Effect of compound **C1·HCl** on CDK9-driven signaling pathways in MV4-11-VR cells after 12 h treatment.

**Supplementary Table 5.** Basic information of the benchmark datasets.

| Datasets | No. of Targets | No. of Drugs | No. of Affinity Pairs | Sparsity |
| --- | --- | --- | --- | --- |
| Davis | 442 | 68 | 30056 | 0% |
| KIBA | 229 | 2111 | 118254 | 24.50% |

**Supplementary Table 6.** The search space for hyperparameters optimization.

| Hyperparameters | Search Space |
| --- | --- |
| Batch Size | {16, 32, 64, **128**} |
| Learining Rate | {0.01, 0.001, **0.0001**} |
| Dropout Rate | {**0**, 0.1, 0.2, 0.3, 0.4, 0.5} |
| Optimizer Type | {SGD, **Adam**, AdaGrad} |
| Drug Embedding Dim | {**512**, 1024, 2048} |
| Target Embedding Dim | {640, 1280, **2560**} |
| Num of GNN Layers | {2, **3**, 4} |
| Num of FC Layers | {2, 3, **4**, 5} |
| num_heads | {1, 2, 4, **8**} |

**Chemistry Methods**

All reagents and solvents were obtained from commercial suppliers and used without additional purification. Anhydrous solvents were purchased from Bidepharm Ltd. (Shanghai, China). Unless otherwise stated, column chromatography was performed on silica gel (200−300 meshes). Reactions were detected by thin-layer chromatography (TLC), and spots were visualized by UV fluorescence (λ_max_ = 254 nm). Nuclear magnetic resonance (NMR) spectral data were recorded on a Bruker Avance AVANCE NEO 500 (500 MHz) or a BRUKER AVANCE NEO Ascend 600 (600 MHz) spectrometer. LCMS (1290 Infinity II-Infinity Lab LC/MSD, Agilent, California, US) and HRMS (Thermo Exactive Plus for ESI) were used for identification of the structures. The purities of the biologically evaluated compounds were >95%.

**General Procedure for the Synthesis of Compounds C1**−**C4**

The synthesis of compounds **C1**−**C4** is outlined in Fig. 3d. First, Suzuki coupling reaction between (4-fluoro-2-methoxyphenyl)boronic acid or (2,4-dimethoxyphenyl)boronic acid and 2-chloro-5-fluoro-4-iodopyridine generated **3a** and **3b**. Subsequently, demethylation of these intermediates was conducted to yield **4a** and **4b**, followed by intramolecular aromatic nucleophilic substitution reaction to produce **5a** and **5b**. Finally, the target compounds **C1**−**C4** were obtained by Buchwald–Hartwig cross-coupling with the corresponding aryl amines.

*2-Chloro-5-fluoro-4-(4-fluoro-2-methoxyphenyl)pyridine* (**3a**). A mixture of 2-chloro-5-fluoro-4-iodopyridine (**1a**, 1.00 g, 3.88 mmol), (4-fluoro-2-methoxyphenyl)boronic acid (**2**, 659.4 mg, 3.88 mmol), Pd(PPh_3_)_4_ (449 mg, 0.38 mmol) in 1,2-ethanediol (10.0 mL) and 2 M aq K_2_CO_3_ (1.60 g K_2_CO_3_ in 5.8 mL water) was degassed using nitrogen. The mixture was then heated at 100°C overnight under a nitrogen atmosphere. After cooling, the water phase was extracted three times using ethyl acetate (EA) (6 mL*3). The combined organic layers were washed with brine (20 mL) and dried over Na_2_SO_4_, concentrated under reduced pressure. The resulting residue was purified by column chromatography (petroleum ether [PE]/dichloromethane [DCM] 4:1–1:1) to afford **3a** as a white power (1.04 g, 96% yield). ^1^H NMR (500 MHz, CDCl_3_) δ 8.27 (s, 1H), 7.36–7.28 (m, 3H), 7.25–7.22 (m, 1H), 6.81–6.70 (m, 2H), 3.82 (s, 3H). LCMS *m/z* (ESI): 256.0 [M+H]^+^.

*2-Chloro-4-(2,4-dimethoxyphenyl)-5-fluoropyridine* (**3b**). Compound **3b** was synthesized in the same way as **3a**. White powder, 1.5 mmol, 364.9 mg, 91% yield. ^1^H NMR (500 MHz, CDCl_3_) δ 8.24 (d, *J* = 1.5 Hz, 1H), 7.35 (d, *J* = 5.2 Hz, 1H), 7.21 (dd, *J* = 8.4, 1.1 Hz, 1H), 6.62–6.55 (m, 2H), 3.87 (s, 3H), 3.82 (s, 3H). LCMS *m/z* (ESI): 268.2 [M+H]^+^.

*2-(2-Chloro-5-fluoropyridin-4-yl)-5-fluorophenol* (**4a**). Boron tribromide solution (1M in DCM, 8 mL) was injected dropwise to the solution of **3a** (1022.0 mg, 4 mmol) in anhydrous DCM (20 mL) at 0°C. The reaction was then warmed to room temperature and maintained for 20 h before being quenched with water (100 mL) at 0 °C. The water phase was extracted with DCM (100 mL*3) three times. The organic layers were washed with brine (200 mL) and dried over Na_2_SO_4_, concentrated under reduced pressure, purified by columnchromatography (PE/EA 10: 0–4: 1) to yield **4a**. White powder, 496.0 mg, 51% yield. ^1^H NMR (500 MHz, CDCl_3_) δ 8.14 (s, 1H), 7.37 (d, *J* = 5.2 Hz, 1H), 7.12 (t, *J* = 7.4 Hz, 1H), 6.57 (dd, *J* = 14.7, 9.2 Hz, 2H). LCMS *m/z* (ESI): 241.9 [M+H]^+^.

*4-(2-Chloro-5-fluoropyridin-4-yl)benzene-1,3-diol* (**4b**)*.* Compound **4b** was synthesized in the same way as **4a**. White powder, 3 mmol, 723.6 mg, crude product. ^1^H NMR (500 MHz, DMSO-*d*_6_) δ 9.88 (s, 1H), 9.72 (s, 1H), 8.40 (d, *J* = 1.6 Hz, 1H), 7.53 (d, *J* = 5.4 Hz, 1H), 7.10 (d, *J* = 8.4 Hz, 1H), 6.44 (d, *J* = 2.3 Hz, 1H), 6.34 (dd, *J* = 8.4, 2.3 Hz, 1H). LCMS *m/z* (ESI): 240.0 [M+H]^+^.

*3-Chloro-7-fluorobenzofuro[2,3-c]pyridine* (**5a**). A solution of **4a** (108.3 mg, 4.5 mmol) and K_2_CO_3_ (1865.8 mg, 13.5 mmol) in NMP was stirred at 140 ^o^C for 4 h. After cooling, water (100 mL) was added to the reaction mixture and a large amount of white solid precipitated. After continuous stirring for 0.5 h, the white solid was filtered, washed with water and dried with an oven to yield **5a**. White powder, 554.0 mg, 56% yield. ^1^H NMR (500 MHz, CDCl_3_) δ 8.71 (s, 1H), 7.95 (dd, *J* = 8.6, 5.3 Hz, 1H), 7.84 (s, 1H), 7.35 (dd, *J* = 8.6, 2.1 Hz, 1H), 7.19 (d, *J* = 2.0 Hz, 1H). LCMS *m/z* (ESI): 221.3 [M+H]^+^.

3-*Chlorobenzofuro[2,3-c]pyridin-7-ol* (**5b**). Compound **5b** was synthesized in the same way as **5a**. White powder, 3 mmol, 329.4 mg, 50% yield. ^1^H NMR (500 MHz, DMSO-*d*_6_) δ 10.49 (s, 1H), 8.15 (d, *J* = 3.3 Hz, 1H), 8.05 (dd, *J* = 8.5, 1.6 Hz, 1H), 7.08 (s, 1H), 6.95 (dd, *J* = 8.6, 1.9 Hz, 1H). LCMS *m/z* (ESI): 220.0 [M+H]^+^.

*7-Fluoro-N-(5-(4-methylpiperazin-1-yl)pyridin-2-yl)benzofuro[2,3-c]pyridin-3-amine* (**C1**). A mixture of intermediate Pd_2_(dba)_3_ (91.6 mg, 0.1 mmol), X-phos (66.7 mg, 0.14 mmol), K_3_PO_4_ (645.9 mg, 3 mmol), **5a** (267.1 mg, 1.2 mmol) and 5-(4-Methylpiperazin-1-yl)pyridin-2-amine (192.3 mg, 1.0 mmol) was degassed using nitrogen. Anhydrous N,N-dimethylformamide (5.0 mL) was then injected dropwise in nitrogen atmosphere. Then the reaction was stirred at 120 °C for 16 h. After cooling, water (40 mL) was added and the water phase was extracted with EA (40 mL*3) three times and washed with brine (100 mL). The organic layers were dried over Na_2_SO_4_, concentrated under reduced pressure to give brown residue. The residue was purified by columnchromatography (DCM/MeOH 10:0–9:1) to yield **C1**. Yellow powder, 307.5 mg, 81% yield. ^1^H NMR (500 MHz, DMSO-*d*_6_) δ 9.48 (s, 1H), 8.64 (s, 1H), 8.42 (s, 1H), 8.20 (dd, *J* = 8.6, 5.6 Hz, 1H), 7.94 (d, *J* = 2.6 Hz, 1H), 7.69 (dd, *J* = 9.3, 2.2 Hz, 1H), 7.41 (dt, *J* = 9.1, 5.9 Hz, 2H), 7.30 (td, *J* = 9.3, 2.3 Hz, 1H), 3.10–3.04 (m, 4H), 2.49–2.45 (m, 4H), 2.23 (s, 3H). ^19^F NMR (471 MHz, DMSO-*d*_6_) δ -109.08. ^13^C NMR (151 MHz, DMSO-*d*_6_) δ 163.26 (d, *J* = 246.1 Hz), 157.51 (d, *J* = 14.5 Hz), 150.52, 148.17, 148.04, 141.15, 134.36, 131.95, 130.46, 126.93, 123.85 (d, *J* = 10.8 Hz), 118.93, 111.90, 111.42 (d, *J* = 24.1 Hz), 100.71, 100.28 (d, *J* = 27.2 Hz), 54.54, 48.96, 45.73. ESI-HRMS *m/z*: [M+H]^+^ calcd for C_21_H_21_FN_5_O, 378.1725; found, 378.1725. HPLC: t_R_ = 6.490 min, 98.60% purity.

Compounds **C2**–**C4** were synthesized by a route similar to that for the synthesis of compound **C1**.

*7-Fluoro-N-(4-(4-methylpiperazin-1-yl)quinazolin-7-yl)benzofuro[2,3-c]pyridin-3-amine* (**C2**). Yellow powder, 0.2 mmol, 35.8 mg, 42% yield. ^1^H NMR (500 MHz, DMSO-*d*_6_) δ 9.77 (s, 1H), 8.78 (s, 1H), 8.50 (s, 1H), 8.33 (d, *J* = 1.6 Hz, 1H), 8.29 (dd, *J* = 8.5, 5.6 Hz, 1H), 7.87 (d, *J* = 9.1 Hz, 1H), 7.73 (dd, *J* = 9.3, 2.0 Hz, 1H), 7.64 (s, 1H), 7.62 (dd, *J* = 9.1, 1.6 Hz, 1H), 7.34 (td, *J* = 9.4, 2.1 Hz, 1H), 3.67 (s, 4H), 2.56 (s, 4H), 2.28 (s, 3H). ^19^F NMR (471 MHz, DMSO-*d*_6_) δ -108.42. ^13^C NMR (151 MHz, DMSO-*d*_6_) δ 163.51 (d, *J* = 246.4 Hz), 163.31, 157.65 (d, *J* = 14.2 Hz), 153.96, 153.13, 150.68, 148.54, 145.77, 132.52, 130.15, 125.71, 124.34 (d, *J* = 11.1 Hz), 118.53, 118.16, 111.56 (d, *J* = 24.0 Hz), 110.13, 109.67, 102.26, 100.35 (d, *J* = 27.3 Hz), 54.42, 48.98, 45.55. ESI-HRMS *m/z*: [M+H]^+^ calcd for C_24_H_22_FN_6_O, 429.1834; found, 429.1833. HPLC: t_R_ = 7.646 min, 95.13% purity.

*7-Fluoro-N-(3-(methylsulfonyl)-5-(trifluoromethyl)phenyl)benzofuro[2,3-c]pyridin-3-amine* (**C3**). Yellow powder, 0.2 mmol, 16.0 mg, 25% yield. ^1^H NMR (500 MHz, DMSO-*d*_6_) δ 10.05 (s, 1H), 8.79 (s, 1H), 8.54 (s, 1H), 8.49 (s, 1H), 8.34 (dd, *J* = 8.6, 5.6 Hz, 1H), 7.74 (dd, *J* = 9.3, 2.3 Hz, 1H), 7.66 (s, 1H), 7.56 (s, 1H), 7.38–7.32 (m, 1H), 3.32–3.32 (m, 3H). ^19^F NMR (471 MHz, DMSO-*d*_6_) δ -61.47, -108.20. ^13^C NMR (151 MHz, DMSO-*d*_6_) δ 163.61 (d, *J* = 246.9 Hz), 157.73 (d, *J* = 14.5 Hz), 150.32, 148.67, 144.06, 142.54, 132.80, 130.61 (d, *J* = 32.6 Hz), 130.09, 124.56 (d, *J* = 10.5 Hz), 123.58 (d, *J* = 272.8 Hz), 118.40, 118.09, 113.50, 111.64 (d, *J* = 24.4 Hz), 102.26, 100.38 (d, *J* = 27.2 Hz), 43.30. ESI-HRMS *m/z*: [M+H]^+^ calcd for C_19_H_13_F_4_N_2_O_3_S, 425.0578; found, 425.0578. HPLC: t_R_ = 13.087 min, 95.85% purity.

*3-((3-(Methylsulfonyl)-5-(trifluoromethyl)phenyl)amino)benzofuro[2,3-c]pyridin-7-ol* (**C4**). Yellow powder, 0.2 mmol, 30.9 mg, 36% yield. ^1^H NMR (500 MHz, DMSO-*d*_6_) δ 10.34 (s, 1H), 9.94 (s, 1H), 8.64 (s, 1H), 8.54 (s, 1H), 8.47 (s, 1H), 8.02 (d, *J* = 8.5 Hz, 1H), 7.63 (s, 1H), 7.40 (s, 1H), 7.03 (d, *J* = 2.0 Hz, 1H), 6.90 (dd, *J* = 8.5, 2.0 Hz, 1H), 3.32 (s, 3H). ^19^F NMR (471 MHz, DMSO-*d*_6_) δ -61.46. ^13^C NMR (126 MHz, DMSO-*d*_6_) δ 160.64, 159.00, 150.06, 148.20, 144.23, 142.50, 133.82, 130.57 (d, *J* = 31.9 Hz), 129.12, 123.63, 123.60 (d, *J* = 272.9 Hz), 118.41, 117.93, 116.48, 113.15, 112.60, 101.26, 98.23, 43.31. ESI-HRMS *m/z*: [M+H]^+^ calcd for C_19_H_14_F_3_N_2_O_4_S, 423.0621; found, 423.0628. HPLC: t_R_ = 11.553 min, 95.83% purity.

*7-Fluoro-N-(5-(4-methylpiperazin-1-yl)pyridin-2-yl)benzofuro[2,3-c]pyridin-3-amine hydrochloride* (**C1·HCl**). A solution of hydrogen chloride solution in 1,4-dioxane (4M, 15 mL) was added to compound **C1** (700 mg) under an ice bath, and the reaction mixture was then stirred at room temperature for 1 h. The progress of the reaction was monitored by TLC. The resultant yellow solid was isolated by washed with MeOH and DCM, affording **C1·HCl** (750.0 mg, 98% yield). ^1^H NMR (500 MHz, DMSO-*d*_6_) δ 12.42 (s, 1H), 11.20 (s, 1H), 8.87 (s, 1H), 8.43 (dd, *J* = 8.4, 5.8 Hz, 1H), 8.18 (d, *J* = 7.9 Hz, 1H), 7.96 (s, 1H), 7.84 (d, *J* = 7.7 Hz, 2H), 7.60 (d, *J* = 9.6 Hz, 1H), 7.42 (t, *J* = 8.2 Hz, 1H), 3.57–3.53 (m, 4H), 3.23–3.16 (m, 4H), 2.82 (d, *J* = 3.8 Hz, 3H). HPLC: t_R_ = 6.357 min, 99.38% purity.

**^1^H, ^19^F, ^13^C NMR, HRMS and HPLC Spectra of Target Compounds.**

*7-Fluoro-N-(5-(4-methylpiperazin-1-yl)pyridin-2-yl)benzofuro[2,3-c]pyridin-3-amine* (**C1**)


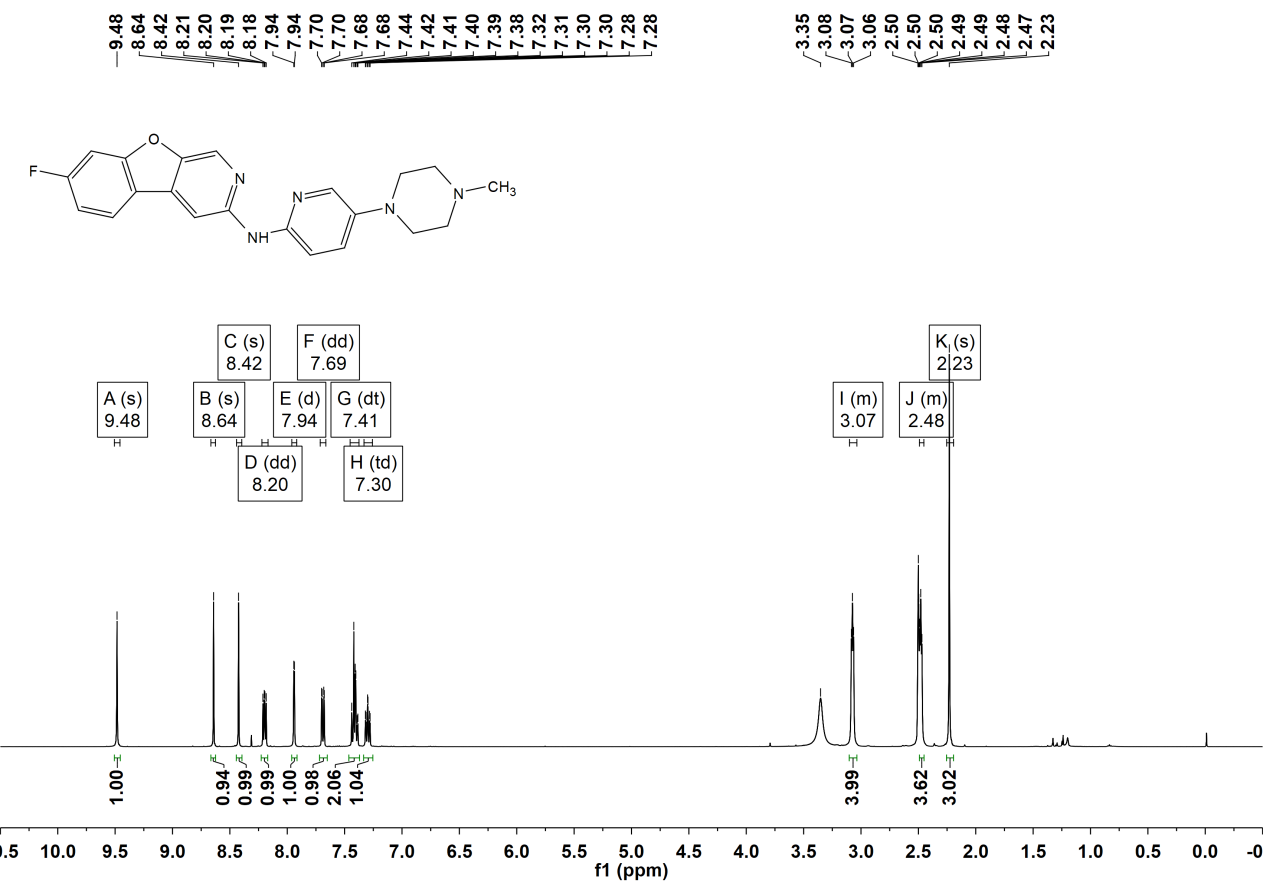


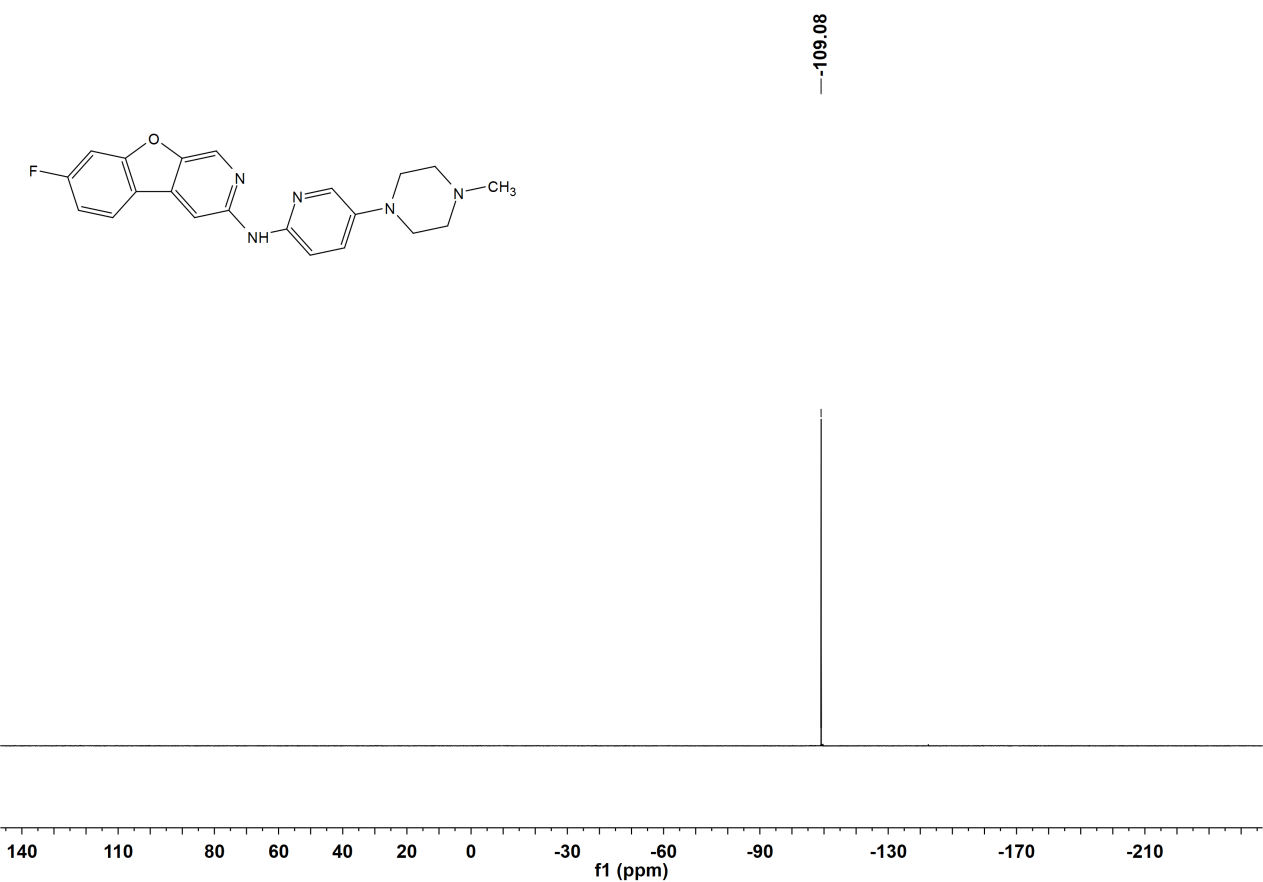


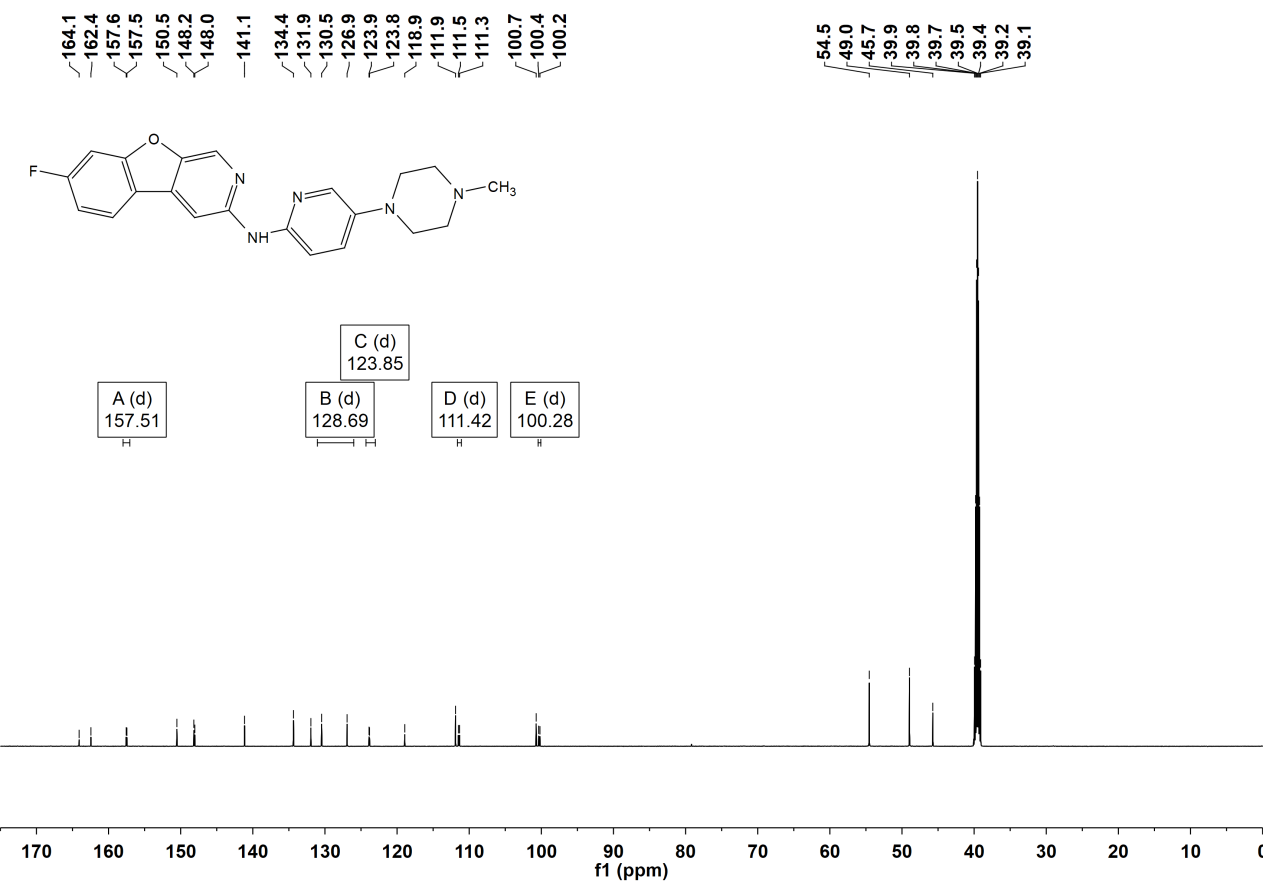

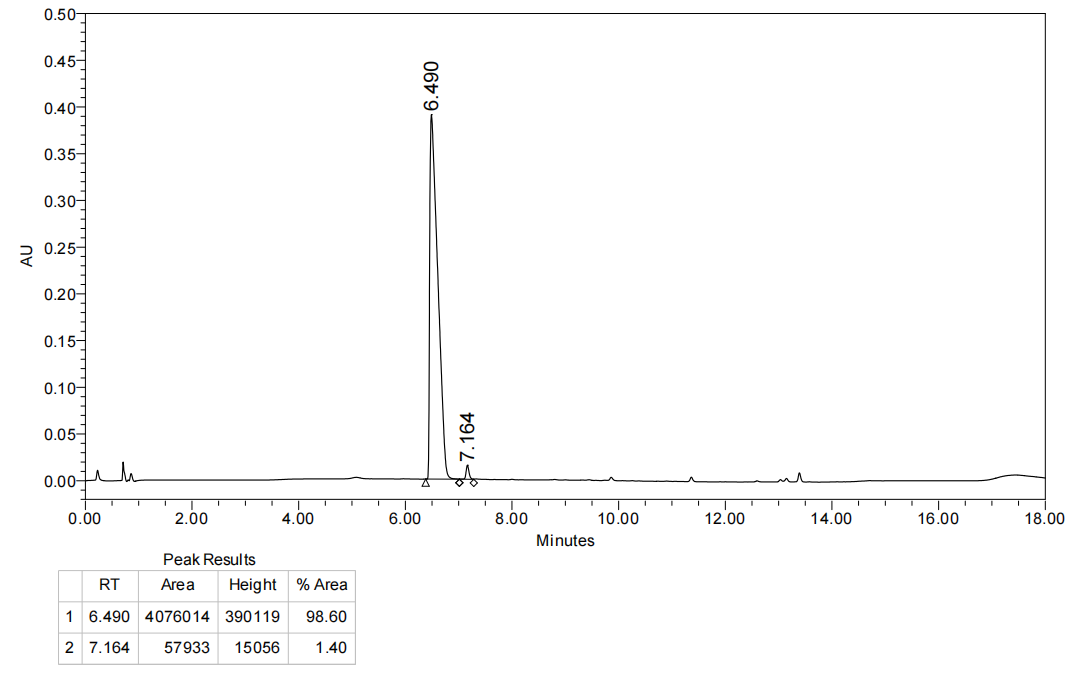


*7-Fluoro-N-(4-(4-methylpiperazin-1-yl)quinazolin-7-yl)benzofuro[2,3-c]pyridin-3-amine* (**C2**)


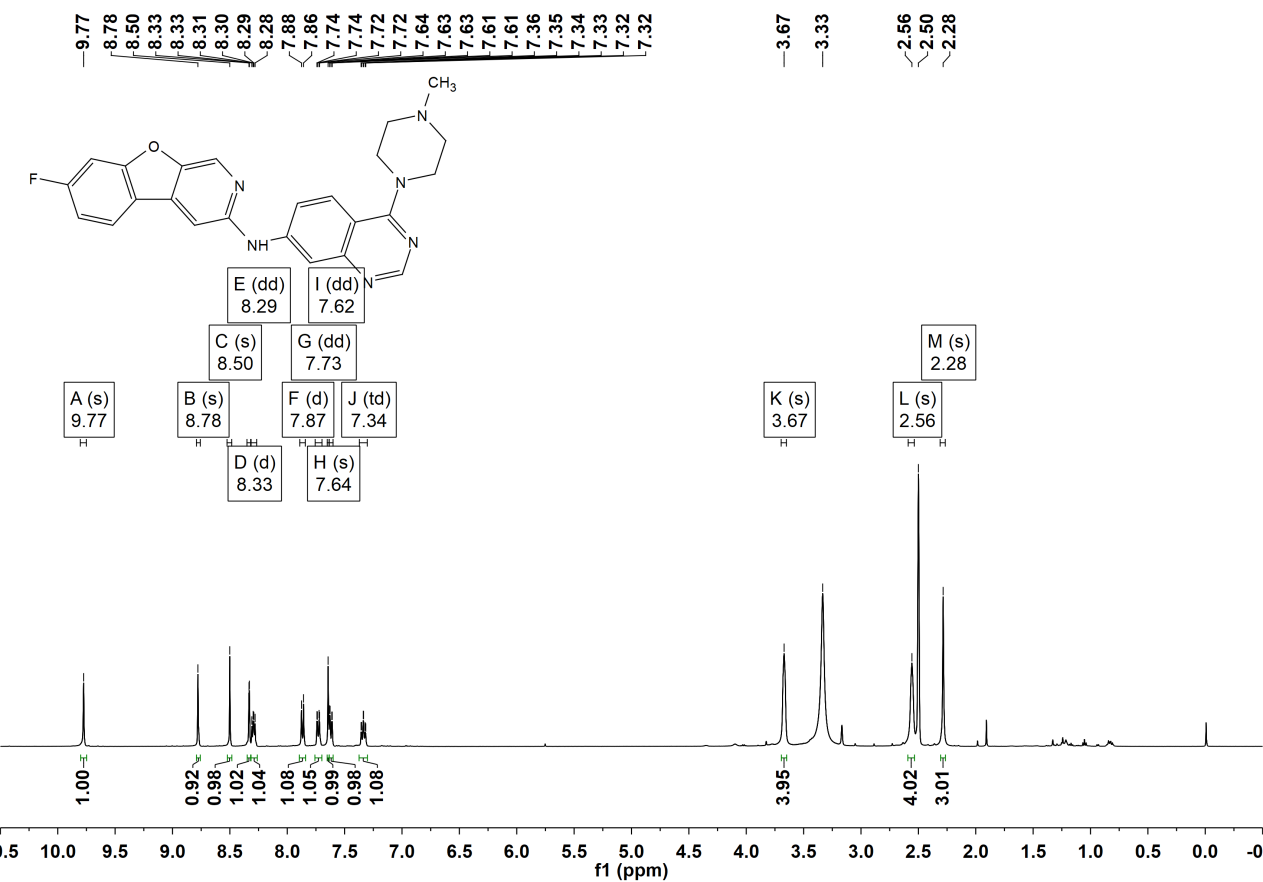


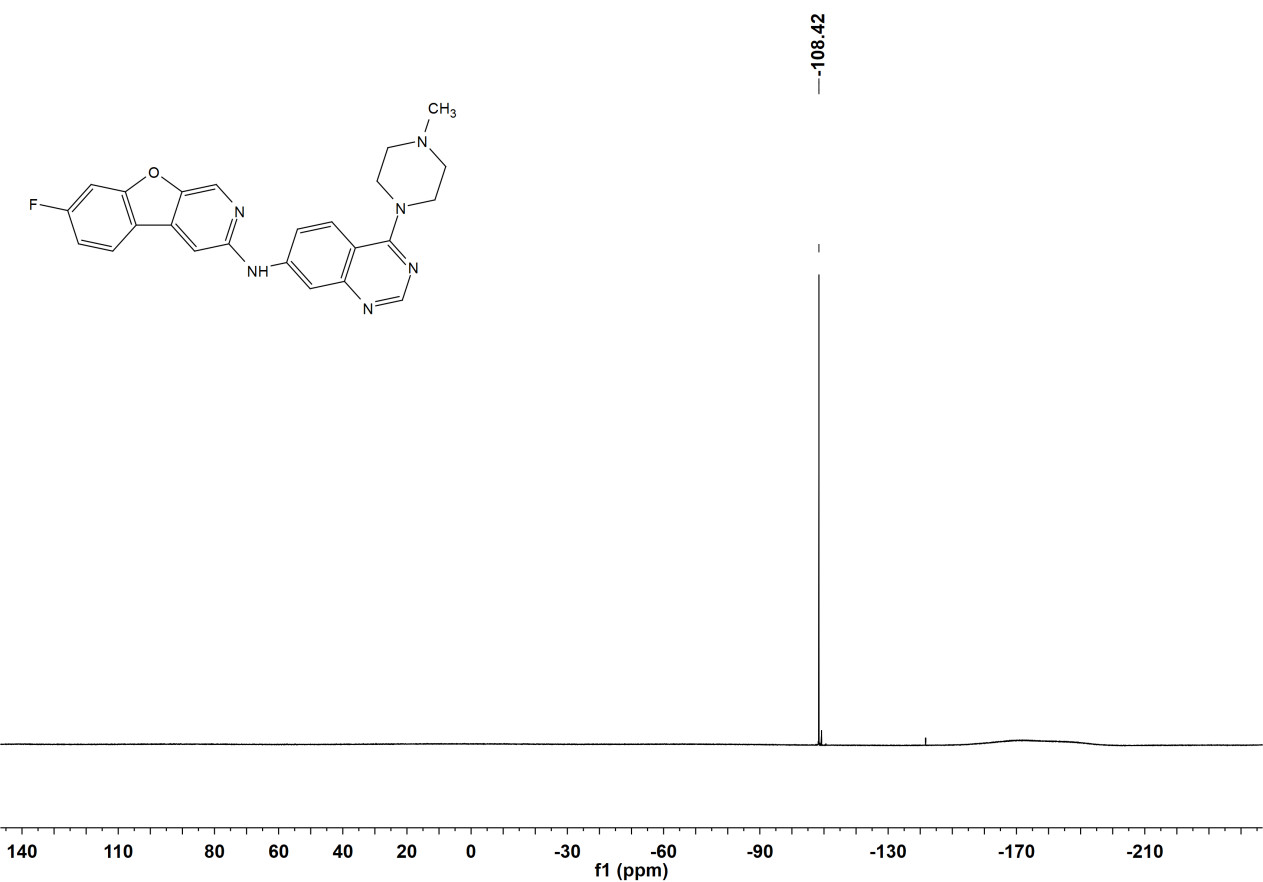


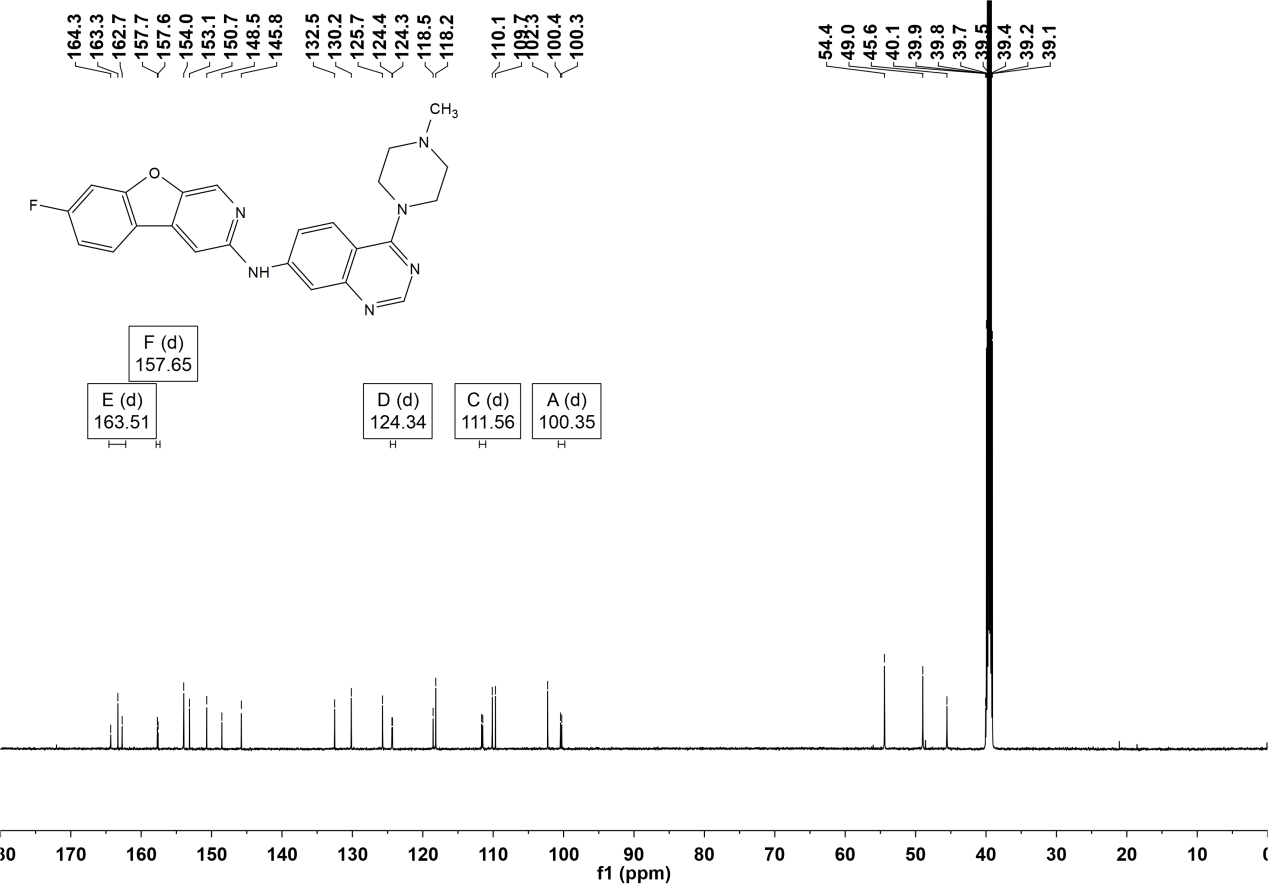

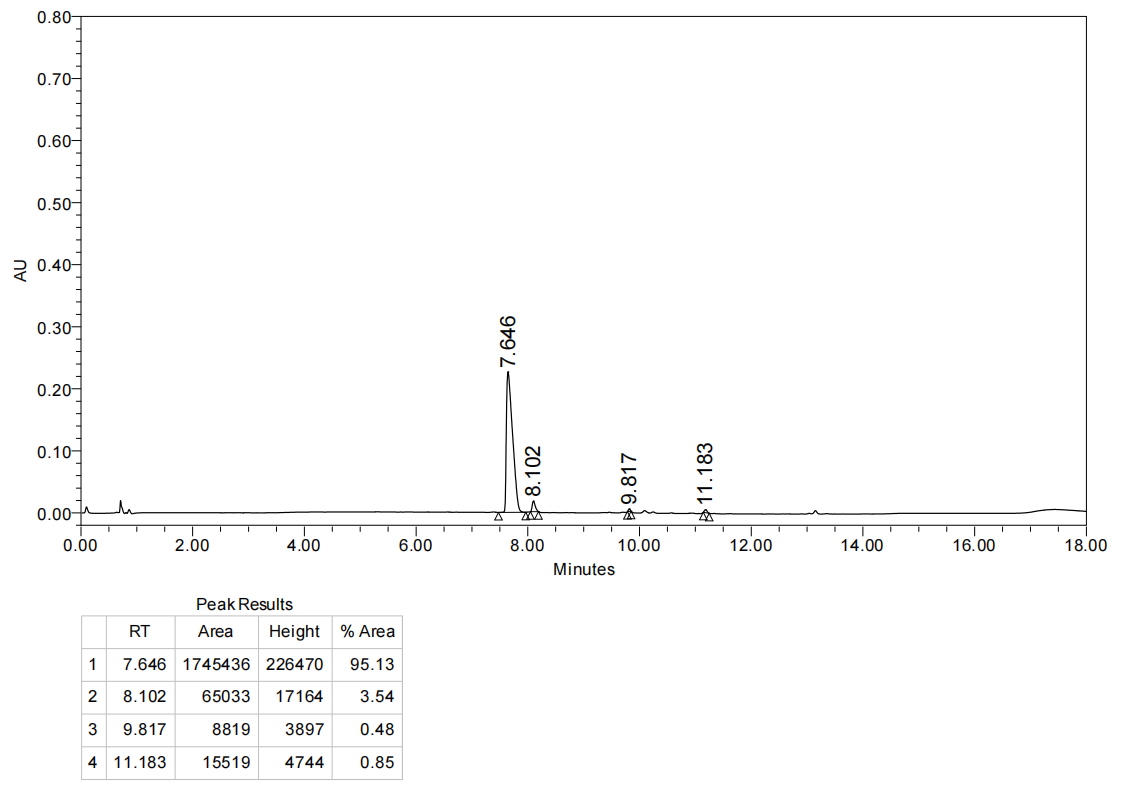


*7-Fluoro-N-(3-(methylsulfonyl)-5-(trifluoromethyl)phenyl)benzofuro[2,3-c]pyridin-3-amine* (**C3**)


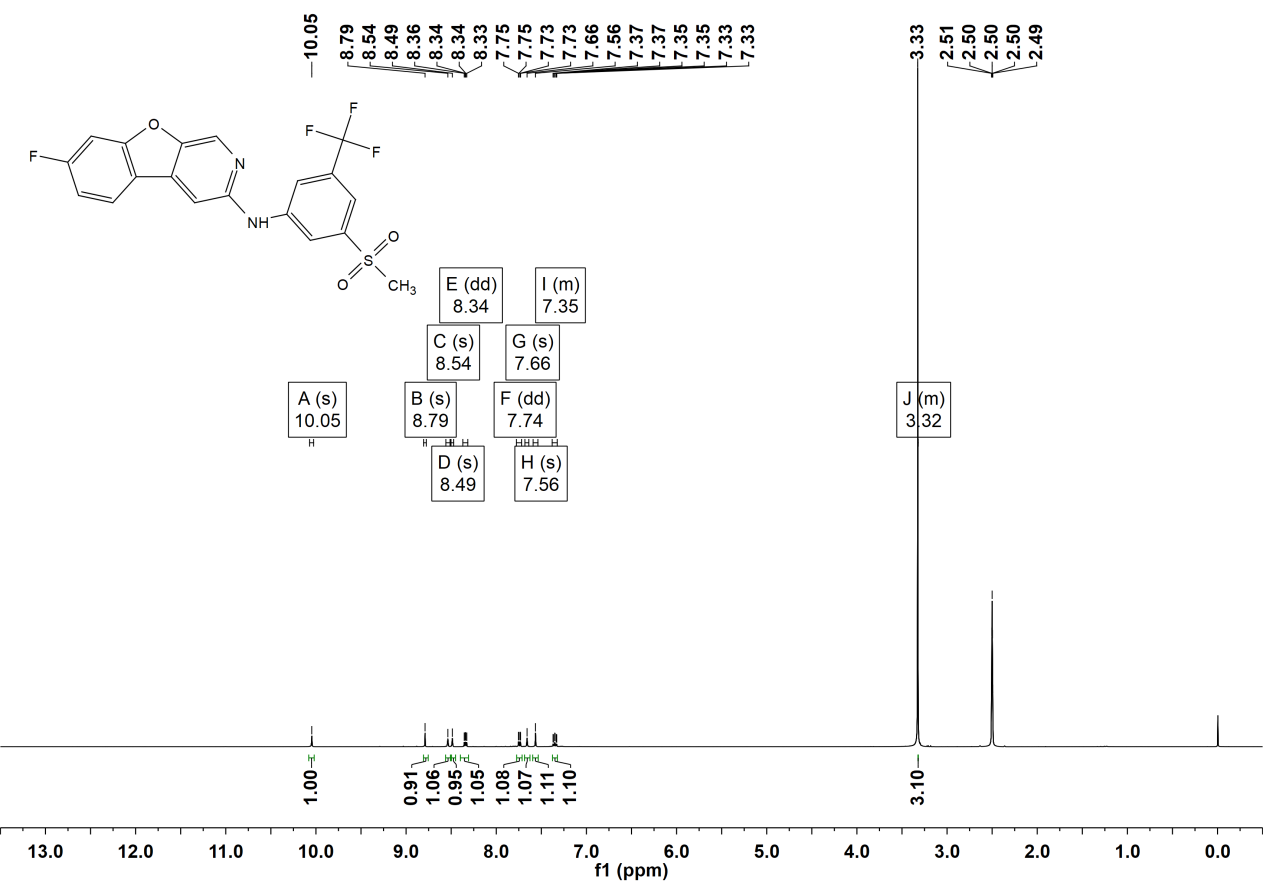


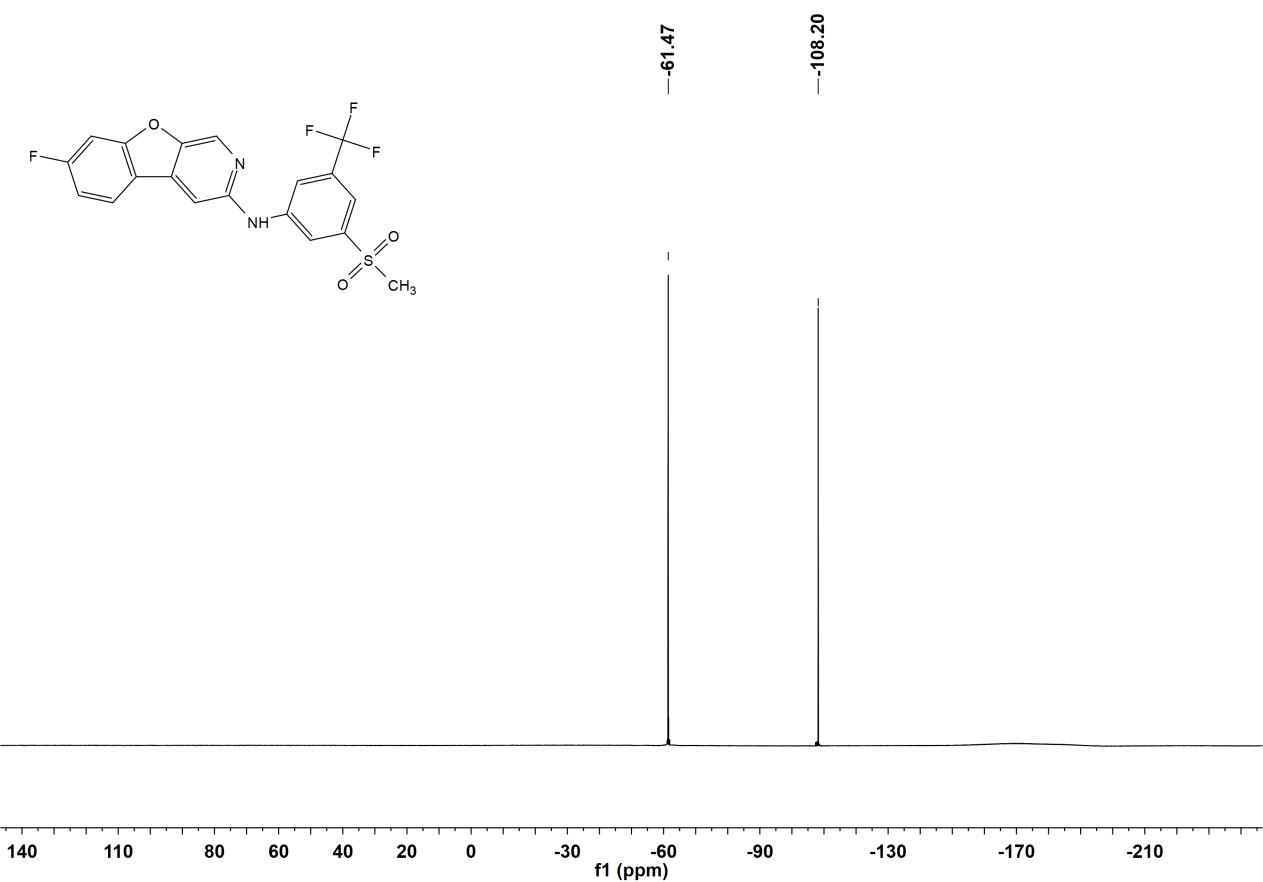


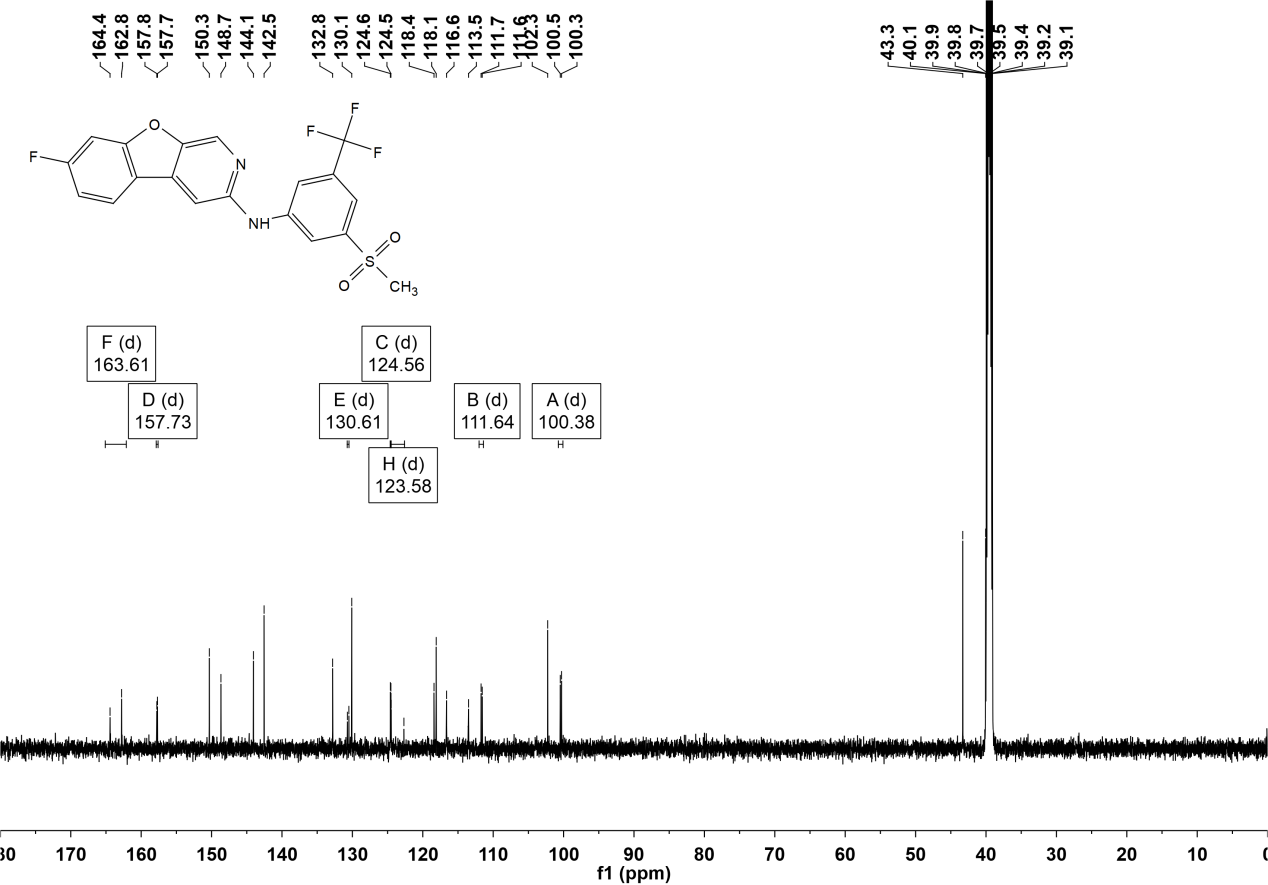

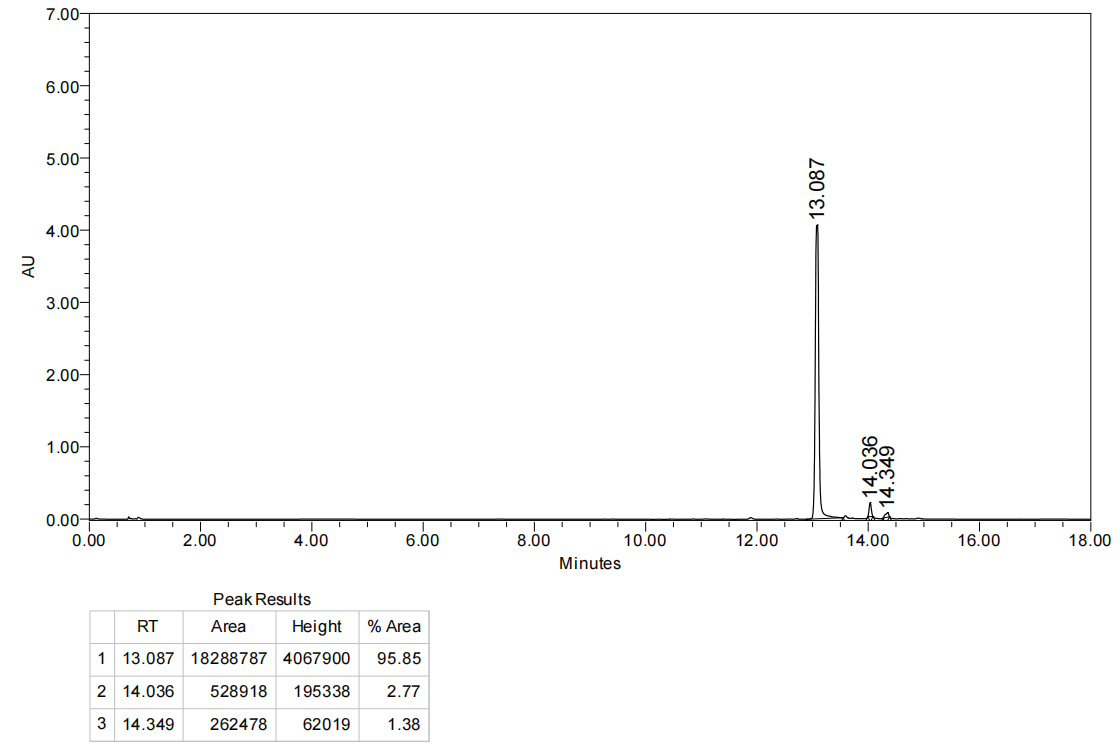


*3-((3-(Methylsulfonyl)-5-(trifluoromethyl)phenyl)amino)benzofuro[2,3-c]pyridin-7-ol (****C4****)*


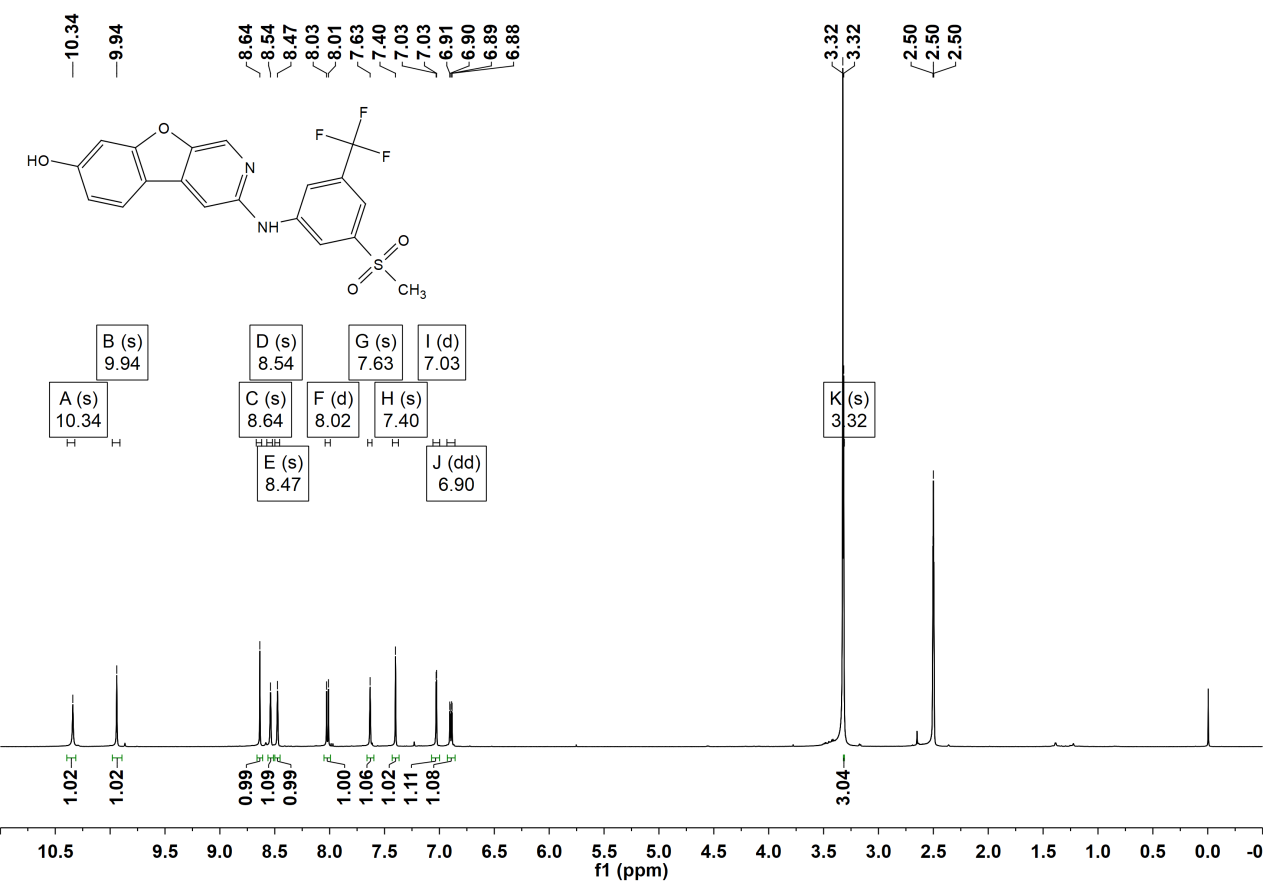


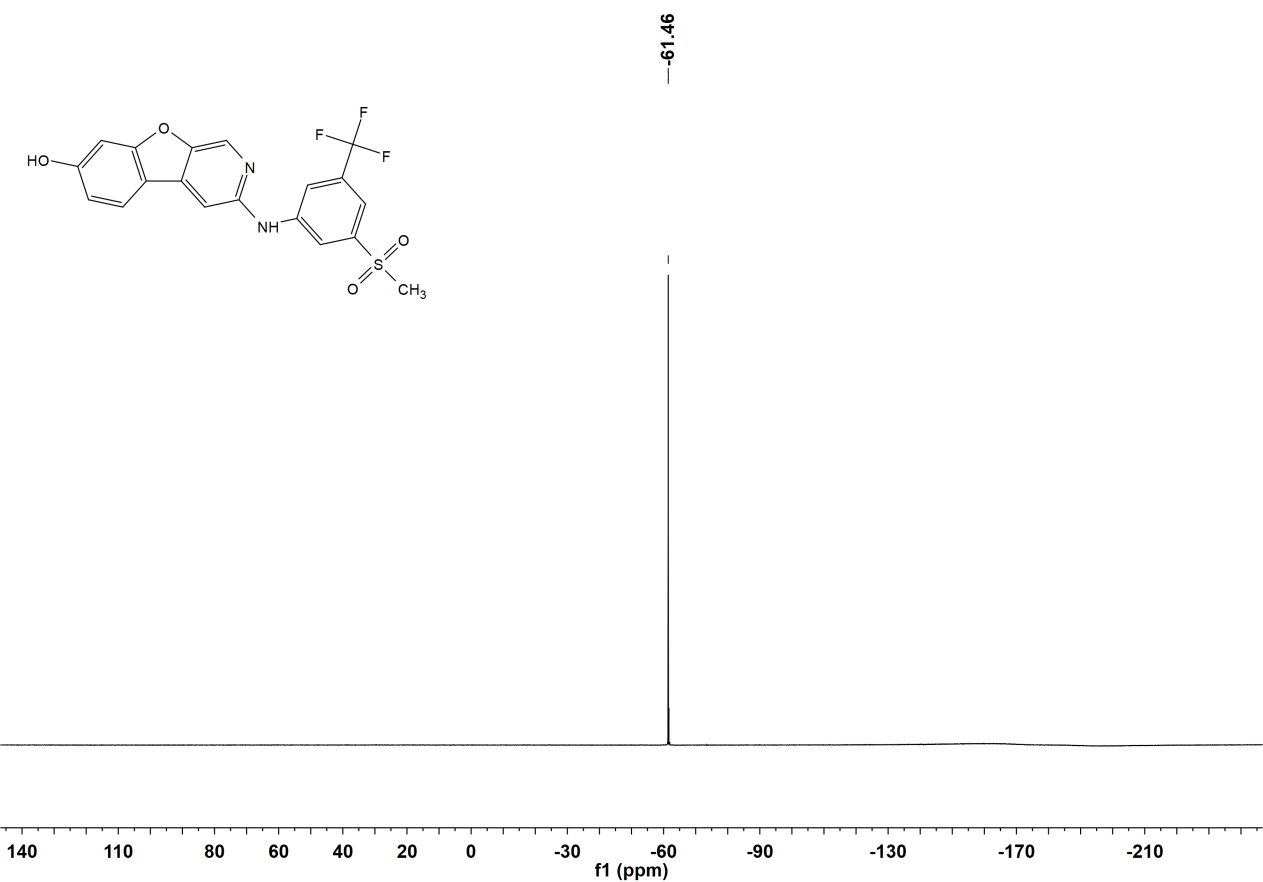


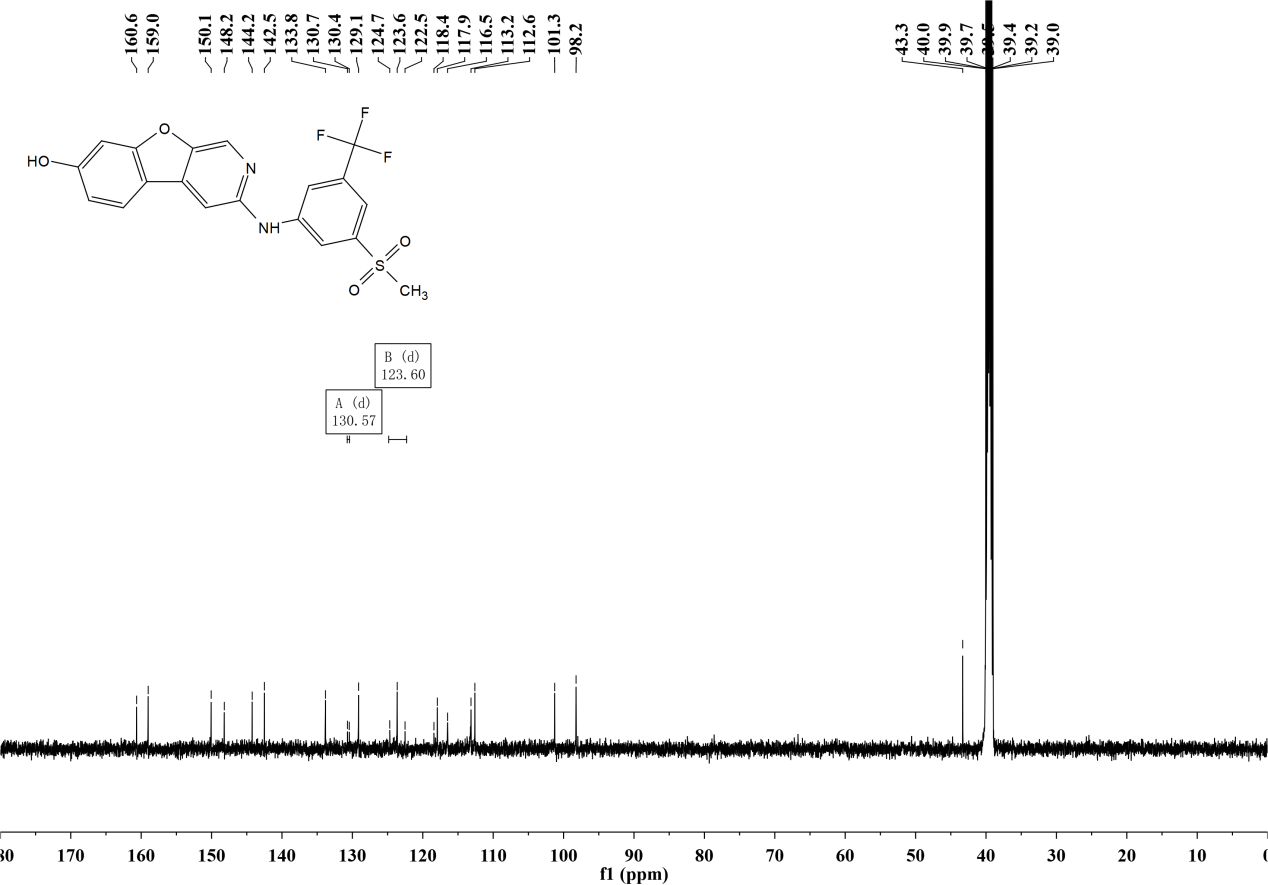

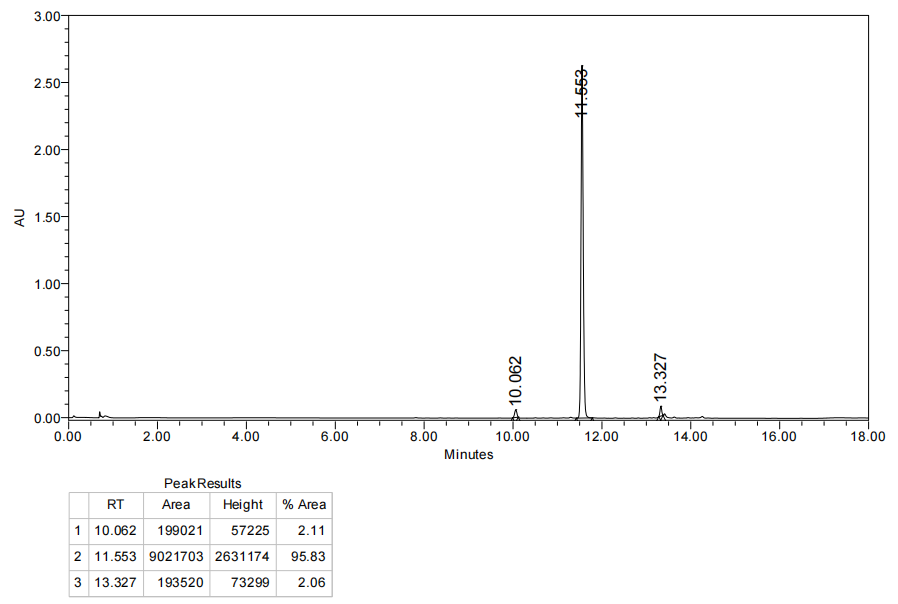


*7-Fluoro-N-(5-(4-methylpiperazin-1-yl)pyridin-2-yl)benzofuro[2,3-c]pyridin-3-amine hydrochloride* (**C1·HCl**)


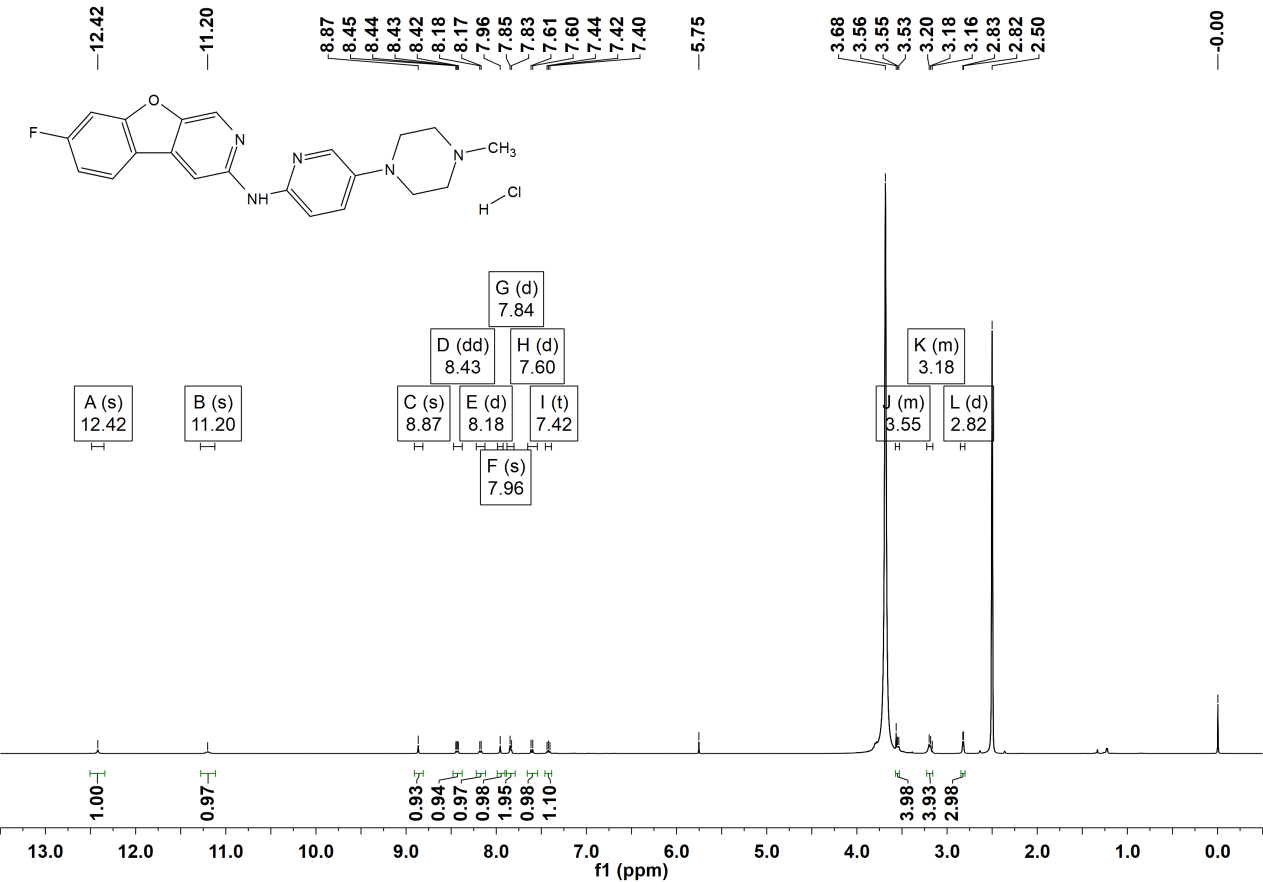


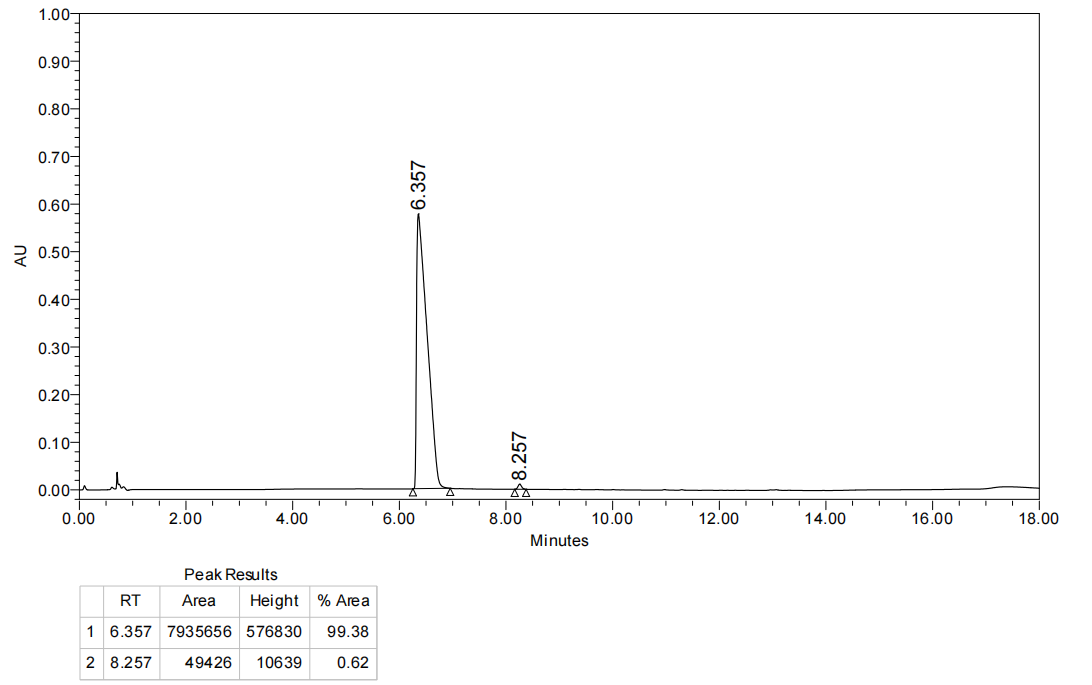

Supplement: Supplementary file 1 — Supporting Information [file ADVS-13-e13099-s001.docx]
